# *In vitro* extinction learning in *Hermissenda*: involvement of conditioned inhibition molecules

**DOI:** 10.3389/fnbeh.2014.00354

**Published:** 2014-10-21

**Authors:** Joel S. Cavallo, Brittany N. Hamilton, Joseph Farley

**Affiliations:** Program in Neuroscience, Department of Psychological and Brain Sciences, Indiana UniversityBloomington, IN, USA

**Keywords:** extinction, memory erasure, *Hermissenda crassicornis*, phosphatases, PP1, PP2B, arachidonic acid, 12(S)-HPETE

## Abstract

Extinction of a conditioned association is typically viewed as the establishment of new learning rather than the erasure of the original memory. However, recent research in the nudibranch, *Hermissenda crassicornis* (*H.c*.) demonstrated that extinction training (using repeated light-alone presentations) given 15 min, but not 23 h, after memory acquisition reversed both the cellular correlates of learning (enhanced Type B cell excitability) and the behavioral changes (reduced phototaxis) produced by associative conditioning (pairings of light, CS, and rotation, US). Here, we investigated the putative molecular signaling pathways that underlie this extinction in *H.c.* by using a novel *in vitro* protocol combined with pharmacological manipulations. After intact *H.c.* received either light-rotation pairings (*Paired*), random presentations of light and rotation (*Random*), or no stimulation (*Untrained*), B cells from isolated CNSs were recorded from during exposure to extinction training consisting of two series of 15 consecutive light-steps (LSs). When *in vitro* extinction was administered shortly (2 h, but not 24 h) after paired training, B cells from *Paired* animals showed progressive and robust declines in spike frequency by the 30th LS, while control cells (*Random* and *Untrained*) did not. We found that several molecules implicated in *H.c.* conditioned inhibitory (CI) learning, protein phosphatase 1 (PP1) and arachidonic acid (AA)/12-lipoxygenase (12-LOX) metabolites, also contributed to the spike frequency decreases produced by *in vitro* extinction. Protein phosphatase 2B (PP2B) also appeared to play a role. Calyculin A (PP1 inhibitor), cyclosporin A (PP2B inhibitor), and baicalein (a 12-LOX inhibitor) all blocked the spike frequency declines in *Paired* B cells produced by 30 LSs. Conversely, injection of catalytically-active PP1 (caPP1) or PP2B (caPP2B) into *Untrained* B cells partially mimicked the spike frequency declines observed in *Paired* cells, as did bath-applied AA, and occluded additional LS-produced reductions in spiking in *Paired* cells.

## Introduction

After an association has been formed between a conditioned stimulus (CS) and an unconditioned stimulus (US), repeated presentations of the CS without the US result in the reduction of the conditioned response (CR), a process known as extinction (Pavlov, 1927). The reduction of the CR produced by extinction training is often only temporary, as evidenced by spontaneous recovery (Reviewed by Myers and Davis, 2002; see Sangha et al., 2003 for spontaneous recovery in the snail *Lymnaea*), or US-induced reinstatement of the extinguished CR (Rescorla and Heth, 1975). The fact that the original associative memory can reappear after extinction training has led to a pervasive view that extinction involves the formation of new fragile, context-dependent learning that counteracts or inhibits the original learning (Rescorla and Cunningham, 1978; Robbins, 1990; Bouton, 1994).

However, recent research has indicated that, under certain conditions, extinction can “erase” the original associative memory, without reappearance of the original CR (Monfils et al., 2009; Schiller et al., 2010). Apparent erasure has also been observed on the cellular level, where extinction training abolished the behavioral effects of associative fear conditioning and reversed conditioning-produced insertion of AMPA GluR1 receptors in mouse (Clem and Huganir, 2010) and rat (Mao et al., 2006) amygdala neurons. In Mao et al. (2006), extinction-produced cellular erasure effects were only observed when extinction was given shortly (1 h, but not 24 h) after the end of learning acquisition, suggesting that erasure might be sensitive to specific acquisition-extinction intervals.

Research in the invertebrate model system *Hermissenda crassicornis* (*H.c.*) has also demonstrated extinction-produced erasure of associative memories on both the behavioral and cellular levels (Richards et al., 1984; Cavallo et al., 2014). Associative memories in *H.c.* are formed using repeated pairings of light (CS) and high-speed rotation (US) (see Farley, 1988b; Crow, 2004; Blackwell and Farley, 2009 for review). Rotation stimulates the *H.c.* vestibular system (statocyst hair cells) and elicits a natural “clinging” response that inhibits locomotion toward light (phototaxis) (Lederhendler et al., 1986). Paired training using light and rotation produces marked suppression of phototactic behavior (CR), which was extinguished using repeated light-alone presentations without any evidence of spontaneous recovery (Richards et al., 1984; Cavallo et al., 2014) or reinstatement (using additional US presentations) (Cavallo et al., 2014) of the CR. Additional neurophysiological data supported the extinction-produced erasure hypothesis and found that extinction reversed conditioning-produced increases in Type B photoreceptor excitability, both in terms of the light response generator potential (Richards et al., 1984) and light-evoked spike frequencies (Cavallo et al., 2014). Because B cells are a principal site of memory storage (Farley and Alkon, 1980, 1982; Richards and Farley, 1987) that are causally related to suppressed phototaxis (Farley et al., 1983), this suggests that the extinction-produced reversal of conditioned behavior results from a corresponding attenuation of enhanced B cell excitability. The goal of the present research was to identify the molecular signaling pathways that mediate extinction-produced alterations in B cell excitability.

Associative conditioning (paired training) increases *H.c.* Type B cell excitability through reductions in somatic K^+^ currents (Alkon et al., 1985; Farley, 1988a; Jin et al., 2009). These alterations are mediated, in part, by training-produced persistent activation of protein kinase C (PKC) (Farley and Auerbach, 1986; Farley and Schuman, 1991). Because PKC-mediated inhibition of K^+^ channels underlies the increased excitability produced by associative conditioning, we hypothesized that extinction training would reverse this process by dephosphorylating K^+^ channels (or channel-associated proteins) through the activation of protein phosphatase 1 (PP1). PP1 constrains learning-produced increases in Type B cell excitability *in vitro* (Huang and Farley, 2001) and has also been implicated as a principal molecule mediating extinction of conditioned taste aversion in mice (Stafstrom-Davis et al., 2001) and rats (Oberbeck et al., 2010). Protein phosphatase 2B (PP2B, aka calcineurin) is an upstream regulator of PP1 (Mulkey et al., 1994) that limits the expression of long-term memories in *Aplysia* (Sharma et al., 2003), constrains contextual fear learning in mice and mediates its extinction (Havekes et al., 2008). PP2B activity is also implicated in the extinction of fear potentiated startle responses in rats (Lin et al., 2003) and in extinction of conditioned taste aversion in mice (Baumgärtel et al., 2008). Therefore, we also examined whether the PP2B-PP1 signaling pathway participated in the extinction changes in B cell excitability. Additionally, because prior *H.c.* work has identified arachidonic acid (AA) and its metabolite 12(S)-hydroperoxy-eicosatetraenoic acid [12(S)-HPETE] as molecules that reduce B cell excitability and enhance K^+^ currents (Walker et al., 2010), we suspected that these molecules might also participate in extinction and decrease B cell excitability, as they do in the related phenomenon of conditioned inhibition (CI) learning (Walker et al., 2010).

To ascertain which molecular mechanisms mediate this process, we developed an *in vitro* protocol. Animals first received paired training (*Paired*), random presentations of light and rotation (*Random*), or no stimulation (*Untrained*). Next, B cells from isolated CNSs were recorded from during exposure to two series of 15 consecutive light-steps (LSs). When this extinction method was administered 2 h (but not 24 h) after paired training, B cells from *Paired* animals showed large and progressive decreases in spike frequency by the 30th LS, while control cells did not. We then combined this protocol with pharmacological manipulations and found that several molecules involved in *H.c.* CI learning also contributed to the spiking decreases produced by *in vitro* extinction, including PP1, PP2B, and AA/12-LOX metabolites.

Finally, these data were incorporated into a conceptual framework to create a molecular model of extinction learning in *H.c.* (**Figure 13**). The key assumptions of this model are: (1) Paired conditioning increases B cell excitability through phosphorylation of somatic K^+^ channels (or associated proteins), (2) *In vitro* extinction (repeated LSs) produces large increases in cytosolic Ca^2+^, but only in paired-trained cells, (3) Large intracellular Ca^2+^ levels preferentially activate PP2B, (4) PP2B disinhibits PP1, (5) PP1 dephosphorylates somatic K^+^ channels (or associated proteins), which reduces B cell excitability, and (6) *In vitro* extinction further reduces B cell excitability through the activation of a parallel AA/12-LOX pathway, which also interacts with somatic K^+^ channels.

## Methods

### Animals

Adult *H.c.* were provided by Monterey Abalone Co. (Monterey, CA) and individually housed in perforated 50-ml plastic tubes in aquaria containing artificial seawater (ASW, Bio-sea Marine Mix, AquaCraft, Hayward, California, pH 7.8–8.2) at 15°C on a 6.5/17.5-h light/dark cycle (4 μ W/cm^2^ radiant intensity), as in Richards et al. (1984). Animals were fed with scallops (*Mytilus edulis*) twice weekly, but food was removed 48 h prior to behavioral testing.

### Animal training

The methods and apparatus used for behavioral training have been described previously (Farley, 1987a) and methods for extinction training were designed after Richards et al. (1984). Animals were placed into clear plastic tubes (20 cm) filled with ASW and mounted on a turntable in a refrigerator (11°C). Vestibular stimulation consisted of high-speed rotation (100 RPM), resulting in a 2.24 *g* centrifugal force stimulation of the statocysts. Photoreceptors were stimulated using whole-field illumination provided by a 60 W incandescent light source (56 μ W/cm^2^ intensity) located above the turntable. Timing and duration of stimuli were controlled automatically using an IBM DOS computer connected to digital/analog controllers made by department engineers. Animals were randomly assigned to one of three treatment conditions: *Untrained*, *Paired*, and *Random*. The *Untrained* animals received no behavioral training and remained in the home aquarium during scheduled training sessions. The *Paired* animals received two consecutive daily sessions of paired conditioning (50 trials each). One trial consisted of paired light and rotation (both 30 s) presentations (simultaneous onsets and offsets), with an inter-trial interval (ITI) of 2 min (variable). *Random* animals received the same type of training as paired conditioning (same amount, stimulus duration, and ITI), but had stimuli presented randomly.

### Nervous-system preparation and intracellular recording

**Figure 1:**
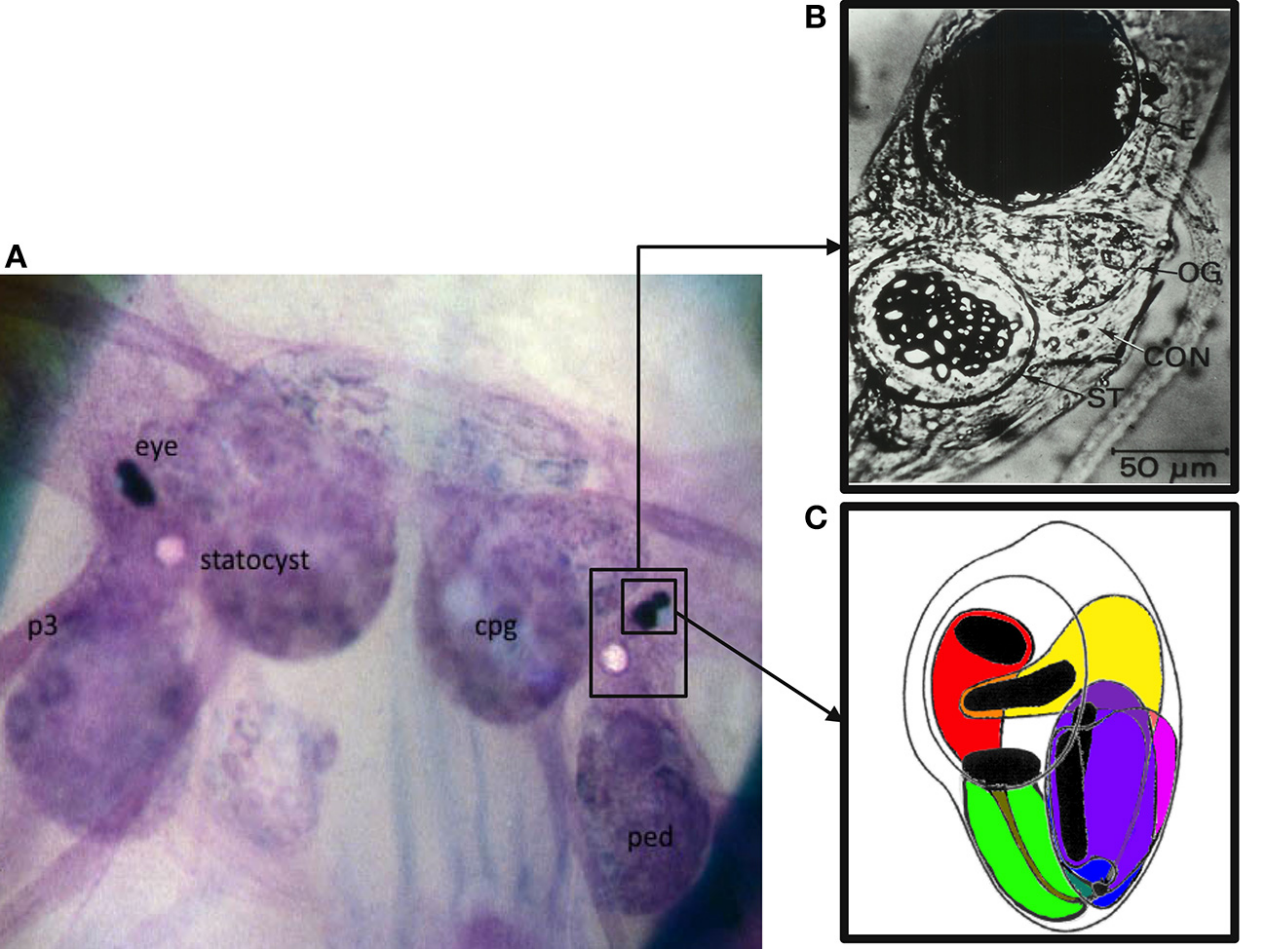
**The *Hermissenda* (*H.c.*) circumesophageal nervous system (CNS): entire CNS, eye, and statocyst. (A)** Low resolution macrophotograph of an *in situ* toluidine-blue stained CNS. Note that the eye and statocyst lie on the dorsal surface of the CNS, in between the cerebropleural (cpg) and pedal (ped) ganglia. Pedal nerves (e.g., p3) project from the pedal ganglia down to the pedal musculature. **(B)** Photomicrograph of a *H.c.* eye (top, dark structure) and statocyst (bottom, ST, crystalline structure). Non-excitable, and non-photoresponsive pigmented cells are responsible for the black appearance of the eye, and obscure the photoreceptors. Portions of the photoreceptors are visible along the bottom of the eye. **(C)** Schematic drawing of a right eye, illustrating the relative positions of the three Type B photoreceptors (medial, intermediate, and lateral, colored here as green, purple, and magenta, respectively) and the two Type A photoreceptors (medial and lateral, colored here as red and yellow, respectively). Note the black rhabdomeric portions of the photoreceptors, where phototransduction occurs. The rhabdomeres are oriented toward the lens. Note also that each photoreceptor has an axo-dendritic process extending from its base. The one extending from the (red) medial A photoreceptor is shown here in gray, running over the soma of the medial Type B photoreceptor. The axo-dendritic processes collect and fasciculate at the base of the eye, to form the optic nerve, which projects underneath the optic ganglion (see **B**) into the cerebropleural ganglia, where it extensively ramifies. Action potentials are generated at the axon hillock region. The majority of fast synaptic potentials (EPSPs and IPSPs) originate distal to the axon hillock at the axo-dendritic ramifications. A variety of slow synaptic responses (e.g., originating from GABA B, muscarinic Ach, and 5-HT GPCRs) are located on the axo-dendritic process and soma. The soma is non-spiking. An appropriately located razor lesion of the axon (optic nerve), close to the base of the eye, eliminates action potentials and fast synaptic potentials, without affecting the light-induced generator potentials, light adaptation, or slow synaptic responses from exogenously applied neurotransmitters. In the synaptically-intact preparation, action potentials recorded from the soma propagate passively and in a retrograde fashion from the axon hillock. Sharp electrode intracellular recordings (and two microelectrode voltage clamp) from the soma allow one to readily record light-induced generator potentials, superimposed action potentials, and synaptic potentials (fast and slow).

General methods for preparing and performing electrophysiological recordings from *H.c.* nervous systems have been described extensively (e.g., Farley and Alkon, 1982, 1987; Farley, 1987a,b, 1988a). Dissected *H.c.* circumesophageal nervous systems (Figure 1) were secured on a microscope slide within a 495 μ L well of ASW (15°C). Each isolated nervous system was incubated in protease (1 mg/ml; Subtilisin A, Sigma P5380) for ~8–9 min at room temperature (~18–20°C) to facilitate cell impalement. After protease exposure, nervous systems were rinsed with ten volumes of cold (4°C) standard ASW.

### *In vitro* extinction protocol

Methods for *in vitro* conditioning using isolated *H.c.* nervous systems have been previously developed for simulating paired conditioning (Farley, 1987b; Farley and Alkon, 1987; Grover and Farley, 1987; Huang and Farley, 2001; Jin et al., 2009). Based on these studies, we developed an *in vitro* extinction protocol that delivered two sets of 15 LSs to isolated *H.c.* CNSs from conditioned or *Untrained* animals. Animals were dissected and standard electrophysiological experiments were performed on Type B photoreceptors (Farley and Alkon, 1982; Farley, 1987b, 1988a). B cells from *Random* animals were recorded from ~1–2 h after training concluded, while *Paired* cells were recorded from ~1–2 h or 24 h after associative conditioning. B cells were impaled and dark-adapted for 15 min, while continuously monitoring spontaneous spiking and synaptic activity. Following dark adaptation, cells were exposed to 15 presentations of a 30 s LS (2 min ISI). These 15 LSs were designed as a replacement for whole-animal extinction training (Richards et al., 1984), which comprises 25 light-alone presentations following paired conditioning. Thus, our *in vitro* extinction method entailed repeated sequential 30 s LS presentations of the same intensity, duration, and frequency as those used during extinction training given to intact animals. After the first 15 LSs, cells were re-dark adapted for 10 min, and given another 15 LSs. The intervening dark adaptation period was administered to minimize light-adaptation effects on B cell excitability that might have accumulated as a result of the first 15 LSs (Farley, 1987a). Light adaptation in invertebrate photoreceptors is a molecular desensitization process that is mechanistically distinct from extinction (Lisman and Brown, 1972; Bader et al., 1976).

### Phosphatase enzymes and inhibitors

Activity of catalytically active protein phosphatase 1 (caPP1) (α-isoform, derived from rabbit skeletal muscle, produced via recombinant *E. coli*, Calbiochem, cat. No. 539493) was 2500 units/mL and was dissolved in buffer to obtain 500 activity units/mL. caPP1 buffer was composed of (in mM): 50 HEPES, 200 NaCl, 1 MnCl_2_, 0.1 EGTA, 2.5 DTT, 0.025% Tween 20 (Polyoxyethylenesorbitan Monolaurate, Sigma P-1379), and 50% glycerol. caPP1 was further diluted in buffer (661.7 nM final concentration), backfilled into microelectrodes, and filled with 1.5 M KCl. caPP1 was introduced into a single B cell via leakage/ionophoresis through the recording electrode using five, 30 s −0.5 nA current injections during the first dark adaptation period.

Catalytically active protein phosphatase 2B (caPP2B) was obtained as a gift from Dr. Claude Klee. Recombinant caPP2B (rCnAB45) (14 μg/ml concentration) was suspended in buffer (in mM): 40 Tris, 100 GuHCl, 100 NaCl, 0.1 CaCl_2_, 2 DTT, and 100 μg/ml soybean inhibitor. caPP2B activity was 15–20 nmol/min, in the assay with 1 nM rCnAB45, in a buffer of (in mM): 40 Tris, 100 NaCl, 0.4 mg/ml BSA, 0.6 CaCl_2_, 0.5 MnCl_2_, and 1 DTT. caPP2B was backfilled into electrodes, filled with 1.5 M KCl, and introduced into B cells using the same methods described for caPP1.

Calyculin A (Sigma-Aldrich #C5552) was dissolved in dimethylsulfoxide (DMSO) (0.1 mM concentration), then diluted with dH_2_O to a stock concentration of 2 μ M. Five microliter of stock solution was added to the ASW bath (20 nM final concentration). Cyclosporin A (Sigma-Aldrich #C3662) was dissolved in 3 M KCl (0.01% DMSO) (100 nM final concentration). Concentrations of calyculin and cyclosporin were 10–20 higher than IC_50_ values reported for inhibition of PP1 (Resjö et al., 1999) and PP2B (Fruman et al., 1992), and identical to concentrations used in prior *H.c.* research (Huang and Farley, 2001).

### Arachidonic acid and metabolite inhibitors

AA (Sigma-Aldrich #A8798) was stored at a stock concentration of 0.1 M in 100% ethanol at −70°C. Daily AA solutions were prepared by diluting stock solutions to 10 mM with dH_2_O. Baicalein (Sigma #465119) was stored in powder form at 4°C and 10 mM solutions were prepared daily in dH_2_O. Final bath concentrations of AA and baicalein were 100 μ M, and ethanol concentrations never exceeded 0.1%.

### Data and statistical analysis

Several measures of B cell excitability were obtained, including: resting membrane potential (V_m_), input resistance (R_in_), and light-evoked spike frequency (number of action potentials, in Hz, during the last 10 s of LS). Input resistance was calculated using Ohms law. Brief (200 ms) current pulses of −0.25 nA were given and voltage drops were recorded. B cells with a V_m_ more positive than −39 mV and a R_in_ of less than 30 MΩ were considered damaged and were discarded.

Differences in B cell spike frequency during LSs 1–2 (averaged) were determined using One-Way ANOVA and Bonferroni *post-hoc* tests. Analysis of LSs 6–30 and LSs 16–30 were done using repeated measures ANOVA comparisons and Bonferroni *post-hoc* pairwise tests. All statistics were performed using the SPSS 20.0 software suite. In cases where a Mauchly's test of sphericity revealed a violation of sphericity (*p* < 0.05), a Greenhouse-Geisser correction was used. Two-tailed significance tests were used (unless otherwise noted), and significant *p*-values are < 0.05.

## Results

Learning-produced alterations in phototactic behavior are correlated with increased light-evoked frequencies in Type B cells (Farley and Alkon, 1982) that drive downstream changes in sensory-interneuron-motoneuron circuits that control locomotor behavior (Goh et al., 1985; Richards and Farley, 1987; Crow, 2004). Behavioral extinction training of intact animals using short acquisition-extinction intervals (15 min) appeared to reverse this cellular correlate of paired conditioning and abolished the learning-produced increases in B cell light-evoked spike frequencies when measured 24 h after conditioning (Cavallo et al., 2014). To investigate the cellular changes that occur during extinction training in real-time, we developed an *in vitro* extinction protocol. Isolated CNSs from associatively trained (*Paired*), or control animals (*Untrained* and *Random*) were exposed to two sets of 15 successive light presentations (light steps, LSs), commencing ~2 h after the conclusion of conditioning. Light-evoked spike activity of Type B photoreceptors was tabulated during each LS. LSs were the same duration (30 s), frequency (2 min ISI), and intensity as LSs given to intact animals during behavioral extinction experiments. Associative conditioning can be simulated in semi-intact preparations or in isolated CNSs using several procedures [e.g., light-B cell depolarization (Farley et al., 1983), light-statocyst hair cell stimulation (Farley and Alkon, 1982, 1987; Farley, 1987b; Grover and Farley, 1987)]. Because these methods produce many of the same learning-produced alterations in B cell excitability and phototactic behavior elicited by paired training *in vivo*, the current procedure consisting of LSs presented to isolated CNSs was used to act as a substitute for whole-animal extinction training (e.g., Richards et al., 1984). We then combined this *in vitro* extinction protocol with selective pharmacological inhibition of the PP1/PP2B phosphatase- and AA/12(S)-HPETE fatty acid-signaling pathways, injection of purified constitutively-active phosphatases, and stimulation with AA. Our results indicated that both phosphatase- and lipid-signaling pathways appeared to be involved in the reductions in B cell excitability produced by *in vitro* extinction.

### *In vitro* extinction produces a progressive decrease in B cell spike frequency, but only in paired-conditioned cells

**Figure 2:**
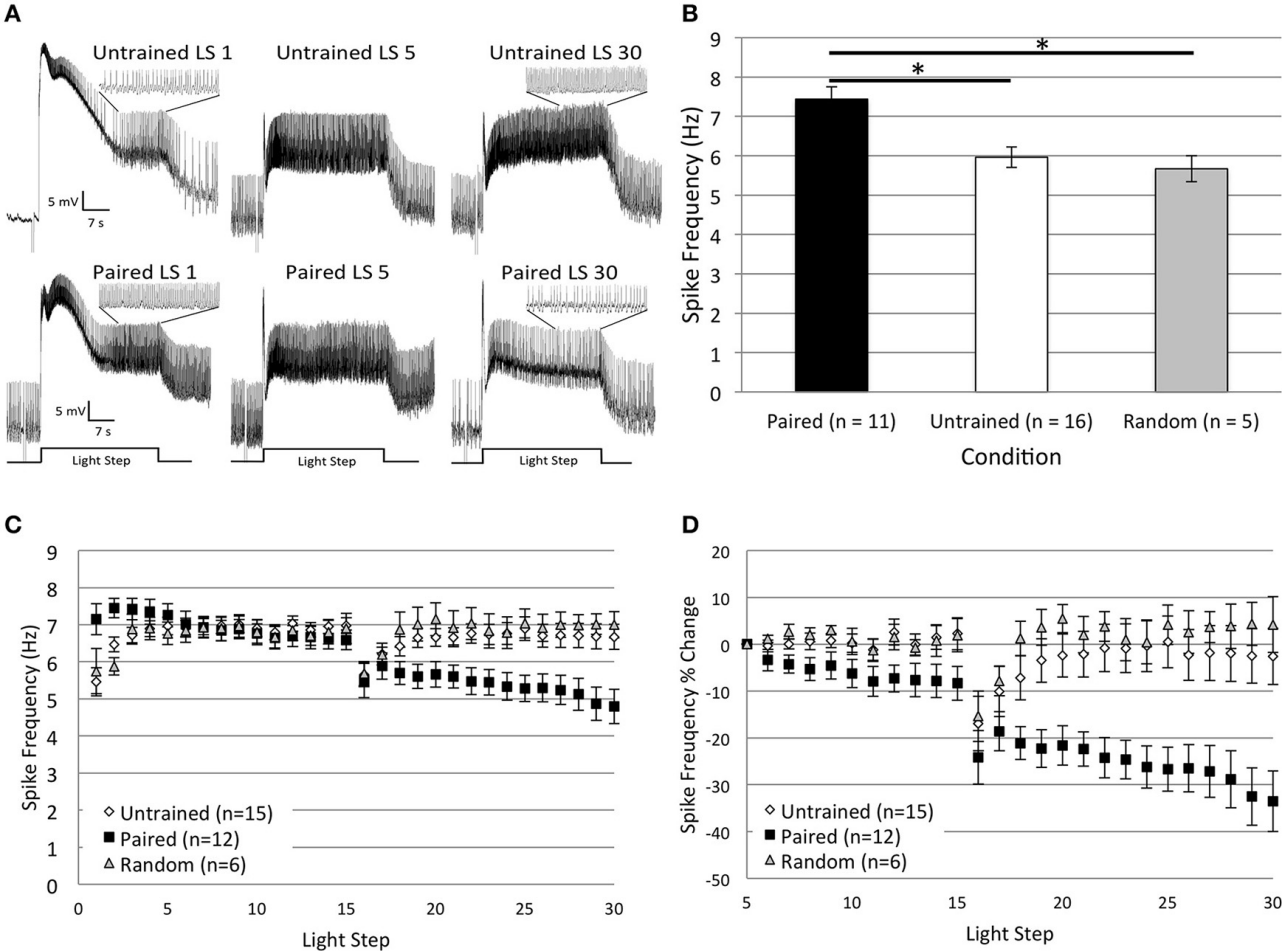
**Thirty repeated light presentations (extinction training) produced a progressive reduction in Type B cell spike frequency, but only in paired-trained cells. (A)** Representative light responses recorded from either untrained (top traces) or paired-trained (bottom traces) Type B photoreceptors from isolated *H.c.* nervous systems. Recordings from *Paired* animals were obtained 2 h following the conclusion of paired conditioning. Light-evoked spike frequencies were recorded during two consecutive sets of 15 repeated light steps (LSs), 2 min inter-stimulus intervals, separated by a 10 min re-dark adaptation period. This procedure served as an *in vitro* simulation of whole-animal extinction training. Spike frequencies were recorded over the last 10 s of each 30 s LS. Inset traces above LSs 1 and 30 for both *Paired* and *Untrained* cells show expanded time scale during last 10 s of LS. Note the more frequent spiking activity in the *Paired* cell during LS 1 compared to the *Untrained* cell, indicative of excitability increases due to associative learning. Both cells had approximately equivalent spike frequencies by LS 5, but after this, the *Paired* cell exhibited a progressive reduction in spike frequency over the course of the next 25 LSs. By LS 30, the *Paired* cell spiked less frequently than the *Untrained* control cell. **(B)** Summary spike frequency data for paired-trained cells and control cells during LSs 1–2 (reported in Cavallo et al., 2014). Paired conditioning increased Type B cell spike frequency during LSs 1–2 above *Untrained* and *Random* (non-associative) control cells, which did not differ. **(C)** Summary spike frequency data for two sets of 15 consecutive LSs. *Paired* cells showed a progressive reduction in spike frequency and exhibited significantly lower spike frequencies during LSs 16–30 compared to *Untrained* and *Random* control cells. **(D)** Summary spike frequency data of the percent change in spike frequency relative to the 5th LS. *Paired* cells showed a steady and significant decrease in spike frequency over the course of 30 repetitive LSs compared to *Untrained* and *Random* cells, which showed very little change. Error bars are ± s.e.m. and significant *p*-values (*p*'s < 0.05) are denoted by an asterisk. Note that the sample size for the *Untrained* (*n* = 15) group was different than what was reported for LSs 1–2 (*n* = 16) because impalement of one cell was lost after LS 24. One *Paired* and one *Random* cell were excluded from LSs 1–2 analysis because electrical noise prevented unambiguous determination of spike frequencies during this period.

As reported in Cavallo et al. (2014, **Figure 7**) and summarized again here in Figures 2A,B, Type B cells from *Paired* (*n* = 11) animals exhibited greater light-evoked spike frequencies during LSs 1–2 (7.44 ± 0.31 Hz) than cells from control animals (*Random*, *n* = 5, 5.67 ± 0.33 Hz; *Untrained*, *n* = 16, 5.96 ± 0.26 Hz) 2 h after the end of associative conditioning. This increased excitability was followed by a large and progressive decline in spiking during later LSs that was unique to *Paired* cells and did not occur in control cells (Figures 2A,C). The initial differences in spike frequencies between *Paired* and control groups diminished with successive LSs (Figure 2C), as observed previously (Farley, 1987a). Spike frequencies were similar for the *Paired* and control groups throughout LSs 4–15. After a re-dark adaptation period (10 min), spike frequencies of these groups began to diverge with successive LSs. *Paired* cells showed a steady decline in spiking during LSs 16–30. A repeated measures ANOVA compared the three groups over LSs 16–30 and revealed that *Paired* (*n* = 12) cells spiked significantly less frequently than *Untrained* (*n* = 15) [*F*_(1, 25)_ = 7.26, *p* = 0.012], and *Random* [*F*_(1, 16)_ = 6.73, *p* = 0.02] cells (Figure 2C), while control cells failed to differ [*F*_(1, 19)_ = 0.20, *p* = 0.659].

Because cells from *Paired* animals initially spiked at higher rates than controls, the decreases in spike frequencies might be more conspicuous for *Paired* cells because of the greater room for decline. Therefore, we also analyzed the relative changes in spike frequency by calculating a percent change score for each cell. The spike frequency of a cell during each LS 6–30 was compared to the spiking during LS 5 for that same cell, when spike frequencies had stabilized and initial training-associated differences were on average no longer apparent. This analysis yielded qualitatively similar results to those reported for absolute spike frequencies. Around LS 6–7, *Paired* cells began to show a progressive decrease in spike frequency that was not evident in control cells (Figure 2D). By the 30th LS, cells from the *Paired* group showed a 33.5 ± 6.5% decrease in spike frequency, which was much larger than the small changes evident in *Untrained* (2.6 ± 6.0% decrease) and *Random* (4.2 ± 7.7% increase) control cells. A repeated measures ANOVA compared the three conditions across LSs 6–30 and revealed that the spike frequency decrease in *Paired* cells was significantly larger than in *Untrained* [*F*_(1, 25)_ = 10.06, *p* = 0.004] and *Random* cells [*F*_(1, 16)_ = 15.08, *p* = 0.001]. *Untrained* and *Random* control cells failed to differ [*F*_(1, 19)_ = 0.26, *p* = 0.618] (Figure 2D). These results indicate that repetitive LSs produced a reduction in B cell spike frequency that was contingent upon prior associative conditioning. These findings paralleled the behavioral *in vivo* extinction results (Figure 7B from Cavallo et al., 2014) and suggest that 30 successive LSs given to isolated *H.c.* CNSs was an effective *in vitro* extinction protocol for producing extinction-correlated reductions in B cell excitability.

### Omission of the dark adaptation period between LSs 15 and 16 does not affect *in vitro* extinction

**Figure 3:**
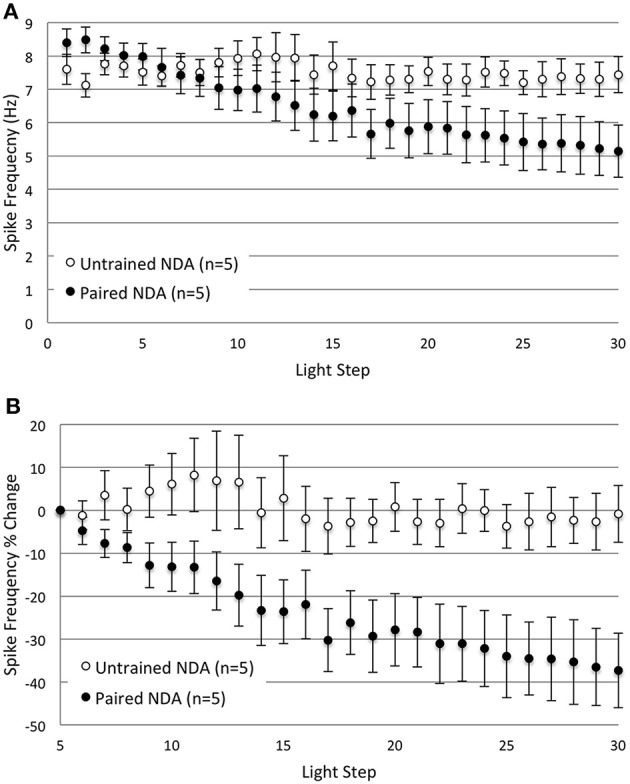
**Omitting the 10 min re-dark adaptation period between LSs 15 and 16 did not substantially alter the decreases in B cell spike frequency produced by repetitive LSs in *Paired* cells. (A)** Summary spike frequency data of Type B cells with no re-dark adaption period (*NDA* groups) in response to 30 successive LSs. *Paired NDA* cells showed a large decrease in spike frequency, while *Untrained NDA* cells showed very little change over the course of 30 LSs. These results were not appreciably different from the spike frequencies observed in their re-dark adapted counter parts (see Figure 2). *Untrained NDA* cells showed little reduction in light-evoked spike frequency over LSs 16–30, while *Paired NDA* cells exhibited a decline that grew significantly larger over time (significant interaction between LS and condition). **(B)** Summary data of the relative percent change in B cell spike frequency from the 5th to 30th LS. *Paired NDA* cells exhibited a significant decline in spike frequencies (~37%) compared to the negligible decrease observed in *Untrained NDA* cells (~1%). Error bars are ± s.e.m. and significant *p*-values are < 0.05. Note that one *Paired NDA* cell was excluded from analysis during LSs 5–30 and LSs 16–30 because impalement was lost after LS 15.

The previous *in vitro* extinction experiments included a 10 min re-dark adaptation period between the two sets of 15 LSs to ensure that any changes in B cell excitability produced by *in vitro* extinction resulted from the extinction process rather than from changes due to light adaptation (see Methods). The question arises as to whether the emergence of the spike frequency differences during LSs 16–30 for *Paired* vs. control cells was predominantly due to: (1) the additional 10 min *per se*, (2) the additional dark adaptation that occurred, (3) the additional LSs, or (4) some combination/interaction of these factors. To address this question, we repeated the *in vitro* extinction experiments for different groups of *Paired* and *Untrained* cells, but omitted the intervening re-dark adaptation period. Thus, cells in each group (*Paired no dark adaptation*, *NDA*, and *Untrained NDA*) were given 30 consecutive LSs with the same LS interval, duration, and intensity used in the prior experiments, but without additional dark adaptation between LSs 15 and 16. Even without the re-dark adaptation period, the same general pattern emerged. Thirty successive LSs produced a progressive reduction in spike frequencies in *Paired*, but not in *Untrained* cells (Figure 3).

*Paired NDA* cells (*n* = 6) showed significantly greater levels of light-evoked spike frequencies than *Untrained NDA* (*n* = 5) cells during LSs 1–2 (8.45 ± 0.27 Hz vs. 7.38 ± 0.34 Hz, respectively) [*t*_(1, 9)_ = 2.48, *p* = 0.035]. During later LSs 16–30, a repeated measures ANOVA found that *Paired NDA* cells (*n* = 5) exhibited a progressive and significant decrease in spike frequency with successive LSs when compared to *Untrained NDA* cells (Figure 3A) [significant interaction between LS and condition: *F*_(14, 112)_ = 4.97, *p* = 0.000]. This indicated that repeated LSs produced a reduction in spike frequency, but only in *Paired* cells.

We next compared *Untrained NDA* and *Paired NDA* groups with their re-dark adapted counterparts and found that they generally did not differ. Spike frequencies of *Untrained NDA* cells during LSs 16–30 did not significantly differ from *Untrained* re-dark adapted cells (*Untrained* group from previous experiment) [*F*_(1, 18)_ = 1.72, *p* = 0.206]. Although a significant interaction between LS and condition was found [*F*_(2.93, 52.79)_ = 2.91, *p* = 0.044, corrected for sphericity], pairwise comparisons of each LS indicated that *Untrained NDA* cells spiked more frequently than *Untrained* re-dark adapted cells, but only at LS 16 (*p* = 0.023). This transient difference in spike frequency was a result of the 10 min dark adaptation period and was gone by LS 17 (see Figure 2C). No other differences were detected during LSs 17–30. Spike frequencies of *Paired NDA* and *Paired* re-dark adapted cells did not differ significantly [*F*_(1, 15)_ = 0.07, *p* = 0.790].

The same pattern and conclusions were obtained when spike frequencies were analyzed as a percent change from the 5th to 30th LS (Figure 3B). *Paired NDA* cells showed a large 37.3 ± 8.7% reduction in spike frequency by the 30th LS that was significantly greater than *Untrained NDA* cells (0.9 ± 6.6% decrease) [main effect of treatment condition, *F*_(1, 8)_ = 6.86, *p* = 0.031] (Figure 3B). Spike frequencies of *Untrained NDA* and *Paired NDA* groups were no different from their re-dark adapted counterparts [*F*_(1, 18)_ = 0.10, *p* = 0.761, and *F*_(1, 15)_ = 1.06, *p* = 0.320, respectively]. These data confirmed that *in vitro* extinction led to a progressive reduction in *Paired* B cell spike frequency that took time and repetitive LSs to develop, regardless of the middle re-dark adaptation period.

### Calyculin a partially blocks the effects of *in vitro* extinction

**Figure 4:**
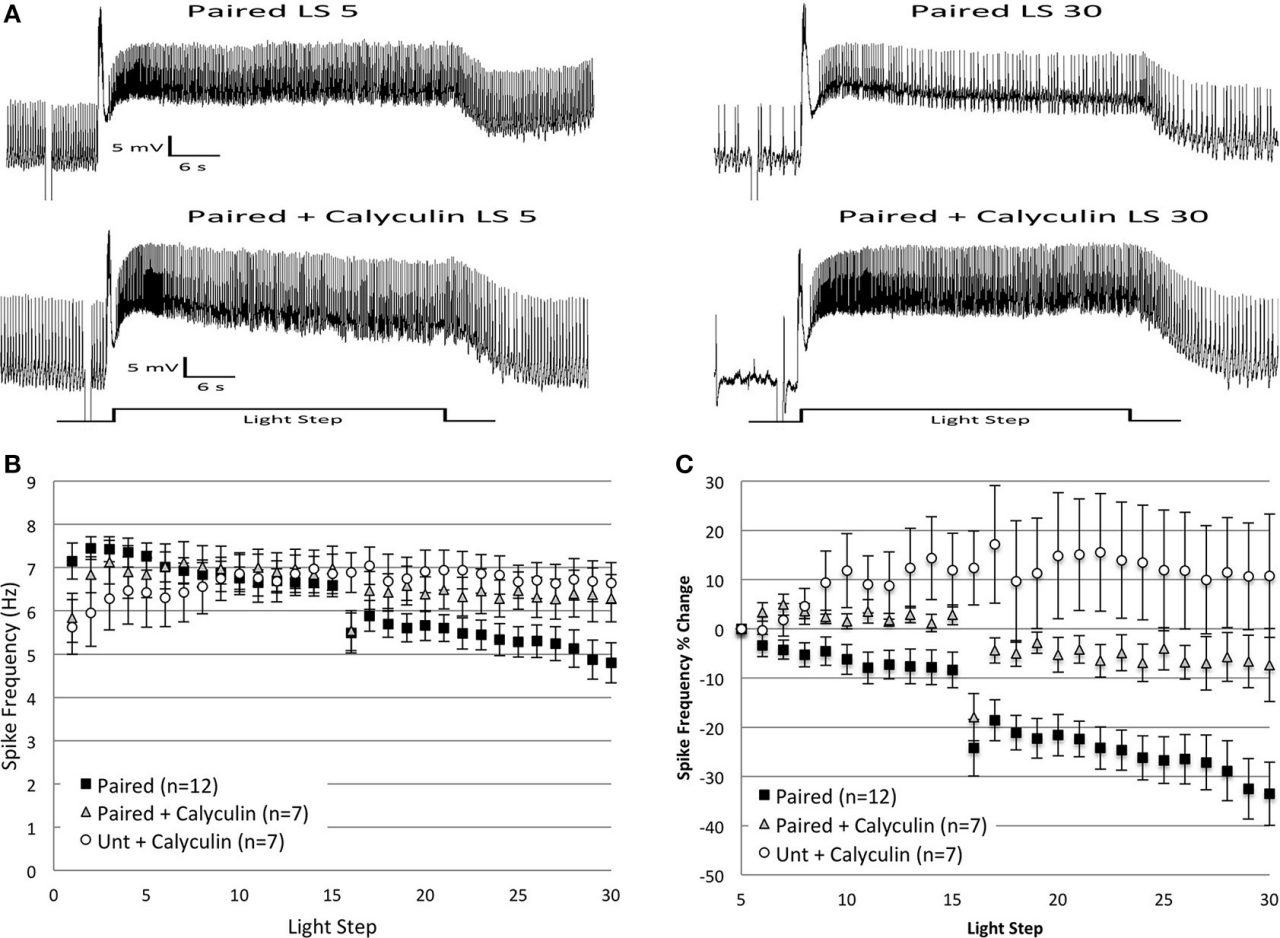
**Bath application of calyculin A (20 nM), an inhibitor of PP1, blocked the reductions in spike frequency produced by repeated LSs in *Paired* cells, when measured as a percent change in spike frequency. (A)** Representative light responses recorded from a *Paired* Type B cell (top trace) or a *Paired* cell incubated in calyculin A (*Paired + Calyculin*; bottom trace) for 15 min prior to *in vitro* extinction. The *Paired* cell showed a significant decline in light-evoked spike frequency (40%) from the 5th to 30th LS, while the *Paired + Calyculin* cell showed a 21% increase. **(B)** Summary spike frequency data of Type B cells exposed to calyculin A. The large reduction in spike frequency observed in *Paired* (no drug) cells during LSs 16–30 was qualitatively smaller when *Paired* cells were incubated in calyculin and was no different than the spike frequencies of *Untrained* cells incubated in calyculin (*Unt + Calyculin*). **(C)** Summary data of the relative percent change in B cell spike frequency from the 5th to 30th LS. The incubation of *Paired* cells in calyculin significantly attenuated the large decrease in spike frequency over the course of 30 LSs normally exhibited by *Paired* cells without drug. *Paired + Calyculin* cells spiked at frequencies not statistically different from *Unt + Calyculin* cells, but frequencies significantly greater than *Paired* (no drug) cells. Error bars are ± s.e.m. and significant *p*-values are < 0.05. Note that one cell in the *Paired + Calyculin* group was omitted from spike frequency analysis during LSs 5–30 and LSs 16–30 because impalement was lost after LS 25.

Prior research found that inhibition of PP1 mimicked and occluded further increases in Type B cell excitability produced by *in vitro* paired conditioning, suggesting that PP1 might limit B cell excitability and oppose the pairing-produced effects of PKC activation (Huang and Farley, 2001). Because inhibitory conditioning (Britton and Farley, 1999) and extinction training produce directionally similar behavioral and electrophysiological effects (enhanced phototaxis, reduced B cell excitability), we hypothesized that PP1 activity might also be recruited during *in vitro* extinction. To test this, we exposed B cells to the PP1 inhibitor, calyculin A, for 15 min prior to *in vitro* extinction training. We predicted that inhibition of PP1 during the course of *in vitro* extinction would block the progressive decrease in spike frequency observed in *Paired* cells, but have little effect in control cells. Consistent with this expectation, *Paired* cells incubated in calyculin showed a much smaller reduction in spike frequency compared to their *Paired* (no drug) counterparts (Figure 4).

Incubation in calyculin did not affect the average spike frequencies of *Paired* (*Paired + Calyculin*, *n* = 8, 6.34 ± 0.47 Hz) or *Untrained* (*Untrained + Calyculin*, *n* = 7, 5.79 ± 0.69 Hz) cells during LSs 1–2 compared to their no drug counterparts. At later LSs 16–30, *Paired + Calyculin* cells (*n* = 7) showed a small reduction in spike frequencies that was not different from *Untrained + Calyculin* cells [*F*_(1, 12)_ = 0.52, *p* = 0.485], nor from *Untrained* (no drug) cells [*F*_(1, 20)_ = 0.25, *p* = 0.624] (Figures 4A,B), indicating that calyculin blocked the large decrease in spike frequency and maintained spike frequencies at control levels. *Paired + Calyculin* cells exhibited spike frequencies that were not significantly greater than *Paired* (no drug) cells during this period [*F*_(1, 17)_ = 2.73, *p* = 0.117].

The effects of calyculin were more apparent when analyzed as a percent change in spike frequency from the 5th to 30th LS (Figure 4C). *Paired + Calyculin* cells showed a negligible 5.6 ± 7.4% decrease in spike frequency over the course of 30 LSs that was not different from *Untrained + Calyculin* cells (10.8 ± 12.5% increase) [*F*_(1, 12)_ = 2.21, *p* = 0.163] (Figure 4C), nor from *Untrained* (no drug) cells (2.6 ± 6.0% decrease) [*F*_(1, 20)_ = 0.02, *p* = 0.893]. In contrast, the 5.6 ± 7.4% decrease in spike frequency observed in *Paired + Calyculin* cells was significantly different from *Paired* (no drug) cells (33.5 ± 6.5% decrease) [*F*_(1, 17)_ = 11.08, *p* = 0.004] (Figure 4C). Thus, pre-incubation of B cells in calyculin prevented the large spike frequency decrease produced by *in vitro* extinction in *Paired* cells.

### Protein phosphatase 1 occludes further spike frequency reductions in *Paired* cells and partially mimics the effects of *in vitro* extinction in *Untrained* cells

To further investigate the involvement of PP1 in the *in vitro* extinction process, we injected the catalytically active subunit of PP1 (caPP1) into B cells prior to the administration of 30 LSs. If *in vitro* extinction activates endogenous PP1, and thereby contributes to the progressive decrease in B cell spike frequencies, then injection of exogenous caPP1 into *Paired* cells should also reduce spike frequencies and occlude any further reductions that result from repetitive LSs (i.e., *in vitro* extinction). Injections of caPP1 into *Untrained* cells might also reduce spike frequencies due to dephosphorylation of spike-regulating K^+^ channels. These reductions might not be apparent immediately, but could develop over time, owing to delays associated with intracellular enzyme diffusion, dephosphorylation rates, etc.

**Figure 5:**
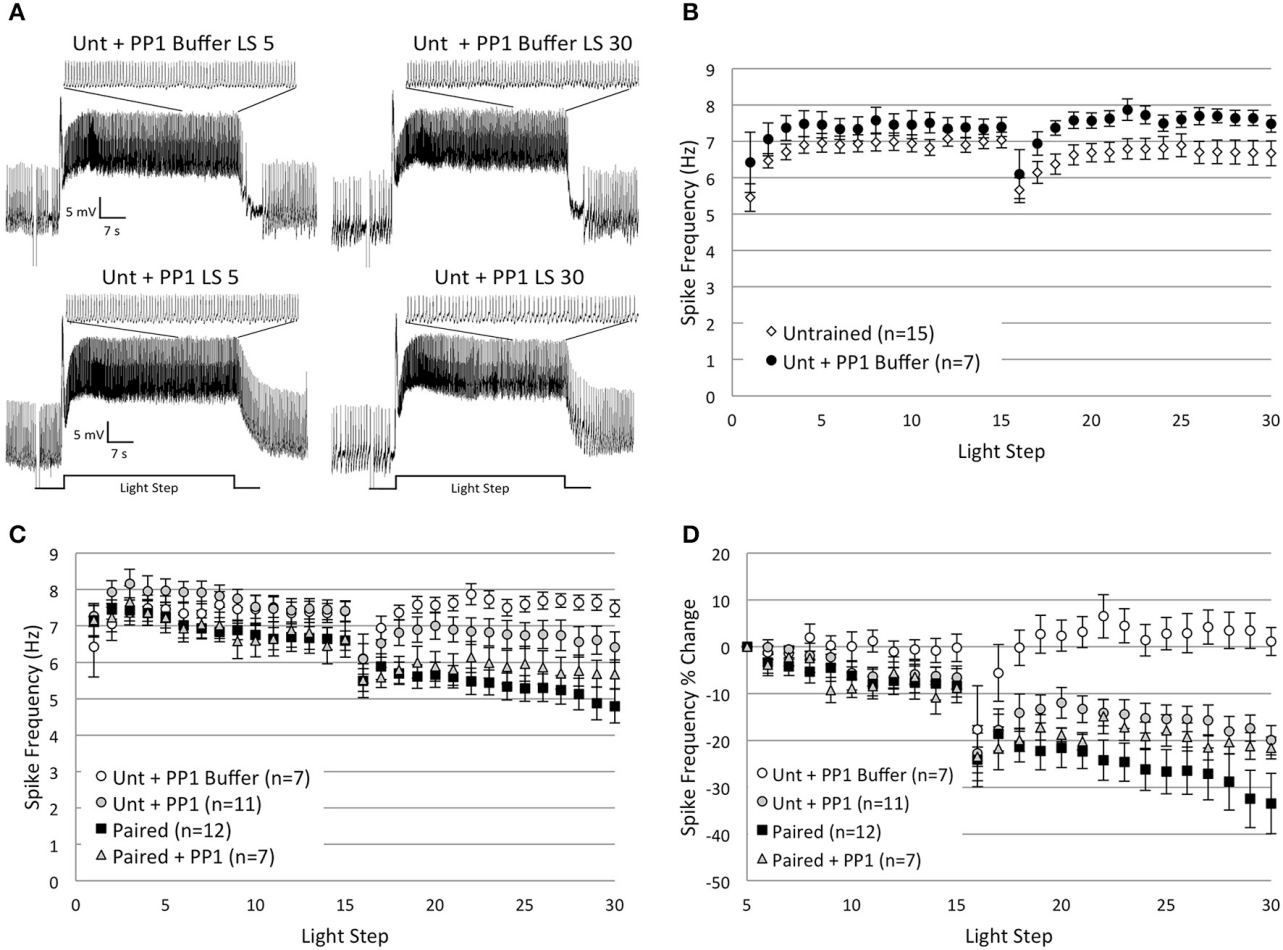
**Injection of catalytically active protein phosphatase 1 (caPP1) reduced spike frequencies in *Untrained* cells by the 30th LS and occluded any additional spike frequency reductions in *Paired* cells. (A)** Representative light responses recorded from an *Untrained* B cell injected with PP1 buffer (*Unt + PP1 Buffer*; top trace) or caPP1 (*Unt + PP1*; bottom trace). The *Unt + PP1 Buffer* control cell showed a slight increase in spike frequency from the 5th to 30th LS, while the *Unt + PP1* cell showed a progressive 23% decrease in spike frequency that mimicked the decreases observed in *Paired* (no drug) cells, i.e., *in vitro* extinction. Inset traces show last 10 s of LS. **(B)** Summary spike frequency data for *Untrained* and *Untrained + PP1 Buffer* control cells across 30 LSs. Note the slight excitatory effect of the buffer. **(C)** Summary spike frequency data for 30 successive LSs indicated that *Unt + PP1* cells showed qualitatively lower spike frequencies during LSs 16–30 compared to *Unt + PP1 Buffer* and *Untrained* controls, and were no different than *Paired* (no drug) cells, thereby producing spike frequency reductions similar to those observed during *in vitro* extinction. *Paired* cells injected with caPP1 (*Paired + PP1*) showed significant decreases in spike frequency below *Unt + PP1 Buffer* control cells. These reductions were no larger than those observed in *Paired* (no drug) cells, suggesting that caPP1 injection occluded any further spike frequency reductions produced by repetitive LSs. **(D)** Summary data of the relative percent change in B cell spike frequency from the 5th to 30th LS. *Unt + PP1* cells exhibited significant declines in spike frequency from the 5th to 30th LS (~20%) compared to *Unt + PP1 Buffer* (1% increase) control cells. These decreases observed in *Unt + PP1* cells were not statistically different than the spike frequency reductions observed in *Paired* (no drug) cells, suggesting that caPP1 is able to produce decreases in spike frequency that are similar in magnitude and time course to the reductions produced by *in vitro* extinction. caPP1 injection into *Paired* cells produced decreases in spike frequency that occluded any further decreases produced by repeated LSs in *Paired* (no drug) cells (~22% vs. ~34%, respectively), suggestive of an occlusion effect. Error bars are ± s.e.m. and significant *p*-values are < 0.05.

In support of the PP1-involvement hypothesis, *Untrained* and *Paired* cells injected with caPP1 exhibited reductions in spike frequency during later LSs 16–30, (Figures 5A,C), an effect more pronounced when measured as a percent change in spike frequency (Figure 5D). The reduction in spike frequency seen in *Paired* cells injected with caPP1 was not greater than what was observed in *Paired* (no drug) cells. Thus, caPP1 injection appeared to partially mimic and occlude the effects of *in vitro* extinction on B cell spike frequency.

An ANOVA of LSs 1–2 compared the average spike frequency of *Untrained* and *Paired* cells with their caPP1-injected counterparts and the *Buffer* control group, and found a main effect of treatment condition [*F*_(4, 47)_ = 4.63, *p* = 0.003]. *Post-hoc* analysis revealed that injection of caPP1 into *Untrained* cells (*Untrained + PP1*; *n* = 11) significantly increased spike frequencies compared to *Untrained* (no drug) cells (7.60 ± 0.29 Hz vs. 5.96 ± 0.26 Hz, respectively) (*p* = 0.005), but not compared to PP1 *Buffer* controls (*n* = 7) (6.74 ± 0.64 Hz) (*p* = 1.0). This indicated that the increased spike frequencies observed in *Untrained + PP1* cells were partly due to non-specific effects of the PP1 buffer. *Paired + PP1* cells (*n* = 7) were no different than *Paired* (no drug) cells during LSs 1–2 (7.19 ± 0.39 Hz vs. 7.44 ± 0.31 Hz, respectively) (*p* = 1.0).

Spike frequencies of *Untrained + PP1* and *Paired + PP1* cells remained approximately at control levels from LSs 4–15 (Figure 5C). During LSs 16–30, *Untrained + PP1* cells showed slight (non-significant) decreases in spike frequencies below *Untrained* [*F*_(1, 24)_ = 0.05, *p* = 0.829] and *Buffer* control [*F*_(1, 16)_ = 2.44, *p* = 0.138] levels, and were significantly higher than *Paired* (no drug) cells [*F*_(1, 21)_ = 6.74, *p* = 0.017] (Figure 5C). *Buffer* control cells showed slight (non-significant) increases in spike frequencies compared to *Untrained* cells [*F*_(1, 20)_ = 3.70, *p* = 0.069] (Figure 5B). This suggests that the small excitatory effects of the PP1 buffer opposed reductions in spike frequencies produced by caPP1 injection, and thereby prevented caPP1 from reducing spike frequencies below *Buffer* or *Untrained* control levels.

*Paired + PP1* cells showed progressive declines in spike frequencies during LSs 16–30 that were no different than the decreases observed in *Paired* (no drug) cells [*F*_(1, 17)_ = 0.61, *p* = 0.446], and were significantly below *Buffer* [*F*_(1, 12)_ = 13.90, *p* = 0.003], but not *Untrained* [*F*_(1, 20)_ = 2.48, *p* = 0.131] cells (Figure 5C). Thus, caPP1 injection into *Paired* cells produced decreases in spike frequencies that were not larger than the decreases observed in *Paired* (no drug) cells, and appeared to occlude the effects of repeated LSs and any further reductions in spike frequency.

To further evaluate the effects of caPP1 injections, we analyzed the relative changes in spike frequencies, measured as a percent change from the 5th to 30th LS. *Untrained + PP1* cells showed a 20.0 ± 3.1% reduction in spike frequency by the 30th LS that was nominally less, but not significantly different, than *Paired* (no drug) cells (33.5 ± 6.5% reduction) [*F*_(1, 21)_ = 2.77, *p* = 0.111] (Figure 5D). This decreased spike frequency of *Untrained + PP1* cells was significantly greater than *Buffer* control cells (1.1 ± 3.0% decrease) [*F*_(1, 16)_ = 11.34, *p* = 0.004], and qualitatively greater than *Untrained* control cells (2.6 ± 6.0% decrease) [*F*_(1, 24)_ = 3.97, *p* = 0.058]. *Paired + PP1* cells showed a significant 21.6 ± 2.4% reduction in spike frequency from the 5th to 30th LS compared to *Buffer* [*F*_(1, 12)_ = 21.55, *p* = 0.001] and *Untrained* [*F*_(1, 20)_ = 5.11, *p* = 0.035] control cells, that occluded any further decreases produced by *in vitro* extinction, and was not significantly different than *Paired* (no drug) cells [*F*_(1, 17)_ = 0.57, *p* = 0.462] (Figure 5D).

In summary, *Untrained* cells injected with caPP1 showed spike frequency reductions that mimicked those observed in *Paired* cells (without caPP1) during *in vitro* extinction. The declines in spike frequencies due to *in vitro* extinction in *Paired* cells injected with caPP1 were no greater than those observed in *Paired* cells (without caPP1), suggesting that caPP1 occluded the spike frequency reductions produced by repeated LSs during *in vitro* extinction. Combined with the calyculin data reported above, these results are consistent with the hypothesis that endogenous PP1 is involved in the *in vitro* extinction-produced changes in B cell excitability.

### Cyclosporin a blocks the effects of *in vitro* extinction

**Figure 6:**
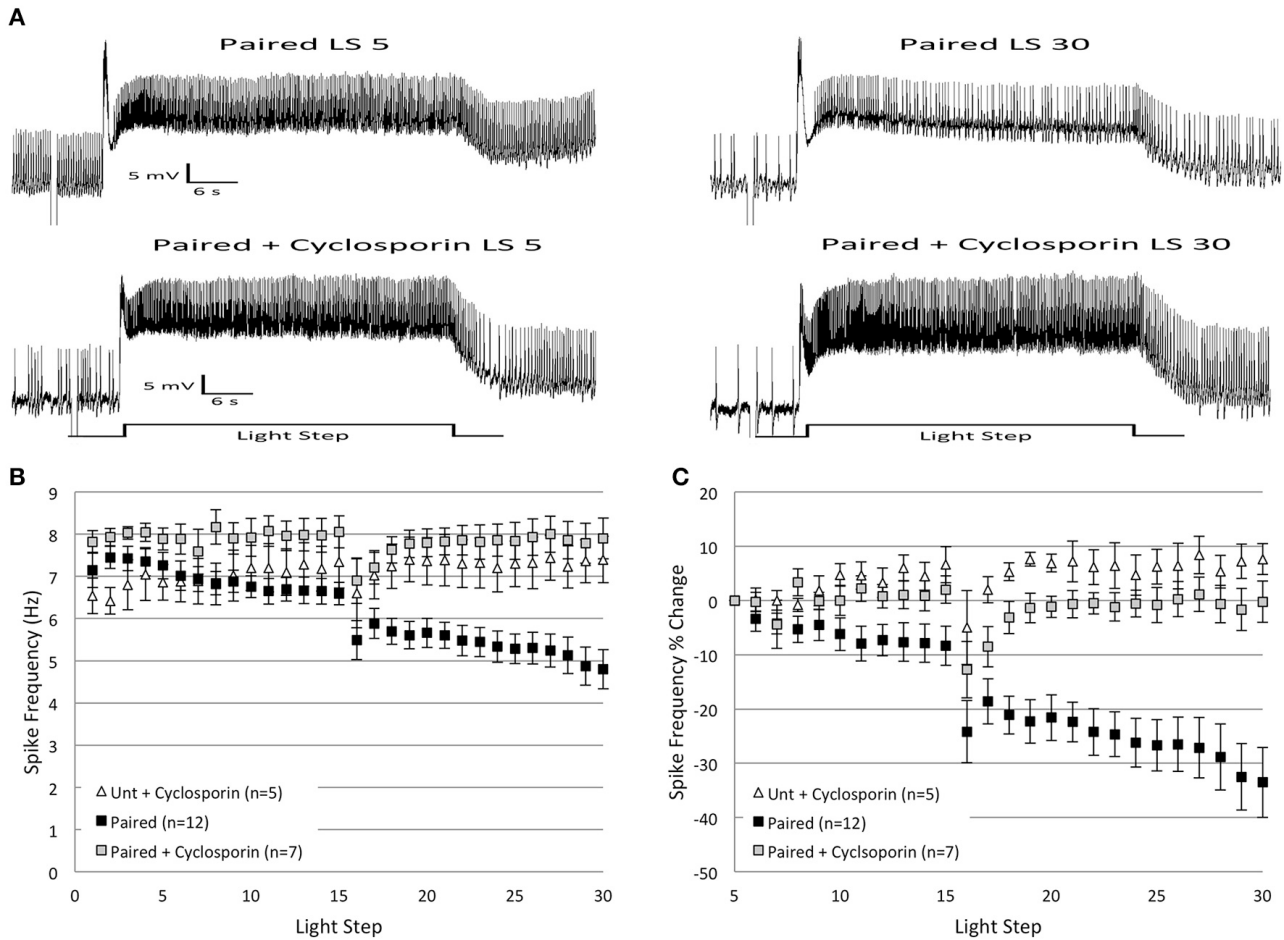
**Injection of cyclosporin A (100 nM in electrode) into *Paired* Type B cells at the start of *in vitro* extinction blocked the progressive reduction in spike frequency produced by repeated LSs in *Paired* cells. (A)** Representative light responses recorded from a *Paired* Type B cell (top trace) and a *Paired* cell incubated in cyclosporin A (*Paired + Cyclosporin*; bottom trace). The *Paired* cell showed a clear decrease in spike frequency from the 5th to 30th LS, while the *Paired + Cyclosporin* cell exhibited no change. **(B)** Summary spike frequency data over 30 LSs of Type B cells injected with cyclosporin. The progressive reduction in spike frequency exhibited by *Paired* (no drug) cells during LSs 16–30 was abolished when *Paired* cells were injected with cyclosporin prior to *in vitro* extinction. Spike frequencies of *Paired + Cyclosporin* cells were no different than *Untrained* cells injected with cyclosporin (*Unt + Cyclosporin*). **(C)** Summary data of the relative percent change in B cell spike frequencies from the 5th to 30th LS. Injection of *Paired* cells with cyclosporin prevented the large decrease in spike frequency over the course of 30 LSs observed in *Paired* cells without drug (0% change vs. ~34% decrease, respectively). The spike frequency changes of *Paired + Cyclosporin* cells over the course of 30 LSs were indistinguishable from those observed in *Unt + Cyclosporin* cells. Error bars are ± s.e.m.

To further probe the link between repeated LSs and PP1 activation, we investigated an upstream regulator of PP1, protein phosphatase 2B (PP2B, aka calcineurin). PP2B is a Ca^2+^/calmodulin-dependent phosphatase (Stemmer and Klee, 1994) that positively regulates PP1 in many cells through the dephosphorylation of inhibitor-1 and the subsequent disinhibition of PP1 (Mulkey et al., 1994). Because repeated LSs can lead to an accumulation of intracellular Ca^2+^ in (untrained) Type B cells, which constrains associative learning in a phosphatase-dependent manner (Muzzio et al., 1999), we hypothesized that the repeated LSs used during *in vitro* extinction would also activate PP2B in *Paired* cells, leading to the disinhibition of PP1 and reductions in B cell spike frequency. To test this hypothesis, B cells from *Paired* or *Untrained* animals were injected with cyclosporin A, a PP2B-specific inhibitor, and then exposed to *in vitro* extinction. If the extinction-produced reduction in B cell spike frequency requires PP2B activity, then the addition of cyclosporin to *Paired* cells prior to *in vitro* extinction should block the decrease. In support of this hypothesis, cyclosporin injection into *Paired* cells blocked the progressive decrease in spike frequency observed during *in vitro* extinction, but had no effect on spike frequency in *Untrained* cells (Figure 6).

Cyclosporin had small (non-significant) facilitatory effects on spike frequencies during LSs 1–2 for both *Untrained* (*Untrained + Cyclosporin*; *n* = 5; 6.48 ± 0.36 Hz) and *Paired* (*Paired + Cyclosporin*; *n* = 7; 7.87 ± 0.23 Hz) cells. This suggests that constitutive activity of endogenous PP2B, and off-target effects of cyclosporin on B cell excitability, were low-to-negligible. During LSs 16–30, cyclosporin blocked the progressive decrease in spike frequencies produced by *in vitro* extinction in *Paired* cells. *Paired + Cyclosporin* cells spiked significantly more frequently than *Paired* (no drug) cells [*F*_(1, 17)_ = 18.35, *p* = 0.001] (Figures 6A,B). The spike frequency of *Paired + Cyclosporin* cells was greater than *Untrained* cells [*F*_(1, 20)_ = 5.12, *p* = 0.035], but no different than *Untrained + Cyclosporin* control cells [*F*_(1, 10)_ = 0.52, *p* = 0.486] (Figure 6B). *Untrained + Cyclosporin* cells were not different from *Untrained* (no drug) cells [*F*_(1, 18)_ = 1.23, *p* = 0.282].

Similar conclusions concerning the effects of cyclosporin followed from analysis of the relative changes in spike frequency from the 5th to 30th LS (Figure 6C). *Paired + Cyclosporin* cells failed to show the progressive declines in spike frequency normally produced without drug, and instead showed a small 0.2 ± 3.8% decline compared to *Paired* (no drug) cells (33.5 ± 6.5% decrease) [*F*_(1, 17)_ = 13.82, *p* = 0.002] (Figure 6C). The negligible spike frequency decrease of *Paired + Cyclosporin* cells was not significantly different from *Untrained* (no drug) cells (2.6 ± 5.98% decrease) [*F*_(1, 20)_ = 0.02, *p* = 0.878], or *Untrained + Cyclosporin* cells (7.6 ± 2.83% increase) [*F*_(1, 10)_ = 3.29, *p* = 0.10] (Figure 6C).

In summary, the injection of cyclosporin into B cells prior to *in vitro* extinction blocked the progressive reduction in B cell spike frequencies produced by repeated LSs, suggesting that PP2B participates in the extinction-produced spike frequency decreases in B cells.

### Protein phosphatase 2B prevents the spike frequency decreases in *Paired* cells due to *in vitro* extinction but mimics the effects of *in vitro* extinction in *Untrained* cells

If endogenous PP2B mediates the decreased B cell spike frequencies observed during *in vitro* extinction, then the injection of exogenous caPP2B into *Paired* cells would be expected to produce a decrease in spiking that occludes any further decreases elicited by the repeated LSs. Prediction of the effects of caPP2B injection into *Untrained* cells is less clear. If PP2B acts exclusively through disinhibition of PP1, but PP1 is either not part of the K^+^ signaling complex and/or the K^+^ channels are not appreciably serine/threonine-phosphorylated in the basal (untrained) state, then injection of caPP2B might not significantly affect spike frequency. Alternatively, if endogenous PP1 is targeted to K^+^ channel signaling complexes and the K^+^ channels are serine/threonine-phosphorylated in *Untrained* cells, then injection of the caPP2B could activate PP1, dephosphorylate K^+^ channels (or associated proteins), enhance K^+^ currents, and decrease spike frequencies similar to that produced by *in vitro* extinction. To test these hypotheses, we injected caPP2B into B cells from *Untrained* or *Paired*-trained animals prior to *in vitro* extinction.

**Figure 7:**
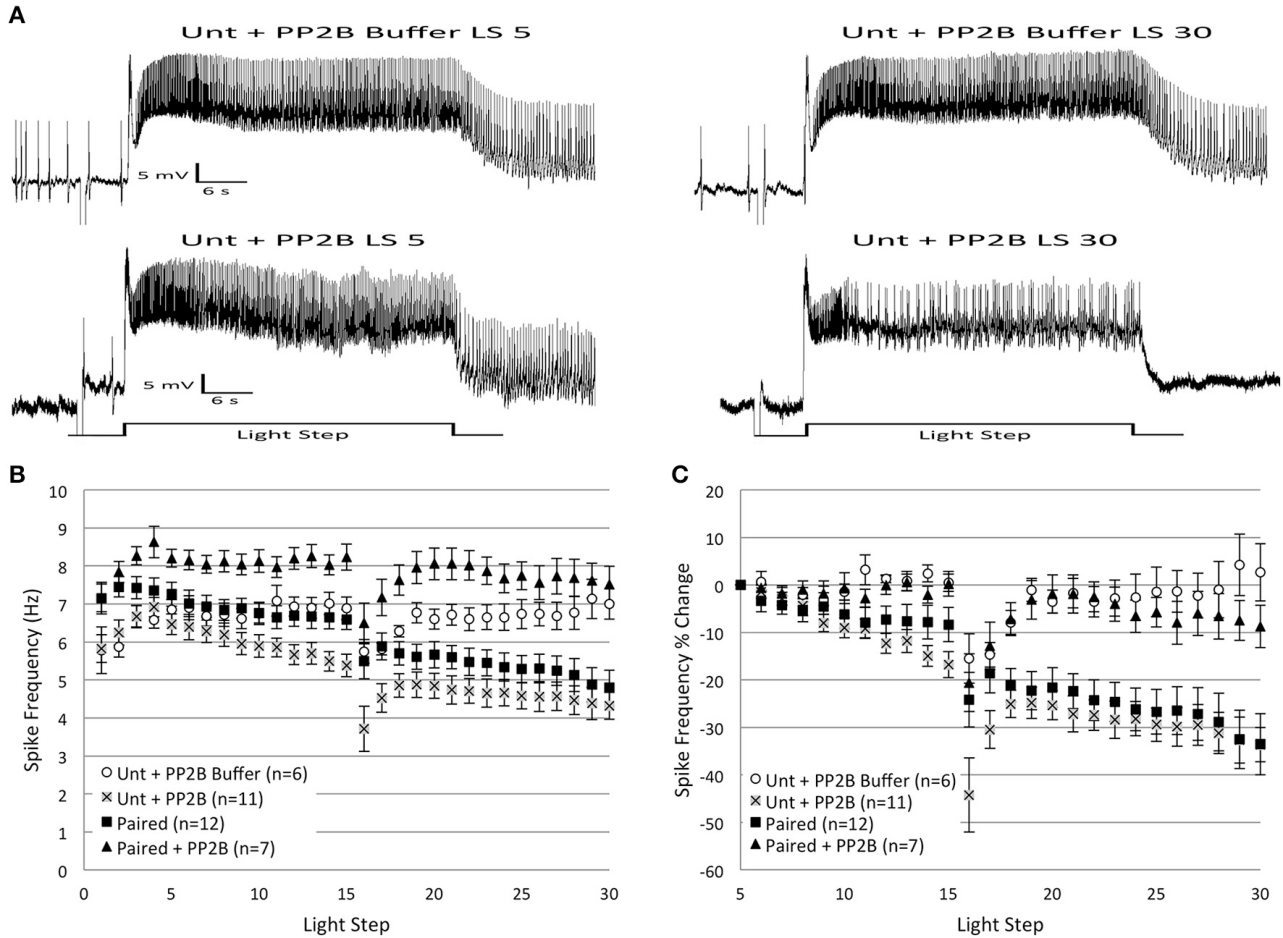
**Injection of catalytically active protein phosphatase 2B (caPP2B) into *Paired* Type B cells prevented the decrease in light-evoked spike frequency produced by repeated LSs, and reduced spike frequencies when injected into *Untrained* cells. (A)** Representative light responses recorded from an *Untrained* B cell injected with PP2B buffer (*Unt + PP2B Buffer*; top trace) or caPP2B (*Unt + PP2B*; bottom trace). The *Unt + Buffer* control cell showed no change in spike frequency from the 5th to 30th LS, while the *Unt + PP2B* cell showed a progressive decrease in spike frequency that mimicked the decreases observed during *in vitro* extinction in *Paired* (no drug) cells. **(B)** Summary spike frequency data across 30 LSs for Type B cells exposed to buffer or caPP2B. *Paired + PP2B* cells did not show decreases in spike frequency and spiked at levels not statistically different from *Unt + PP2B Buffer* control cells. In contrast, *Unt + PP2B* cells showed significantly lower spike frequencies during LSs 16–30 compared to *Unt + PP2B Buffer* controls, and were no different than *Paired* (no drug) cells. **(C)** Summary data of the relative percent change in B cell spike frequencies from the 5th to 30th LS. Injection of caPP2B into *Paired* cells blocked the large reduction in spike frequency observed in *Paired* (no drug) cells; *Paired + PP2B* cells showed significantly smaller decreases in spike frequencies (~9%) over the course of 30 LSs. *Unt + PP2B* cells exhibited significant decreases in spike frequencies from the 5th to 30th LS (~34%), which mimicked the substantial reductions produced by repeated LSs in *Paired* (no drug) cells (~34%), except that they occurred sooner. Error bars are ± s.e.m. and significant *p*-values are < 0.05.

Counter to our expectations, caPP2B injection into *Paired* cells did not decrease spike frequencies, but instead prevented the reduction in spiking normally produced by *in vitro* extinction (Figure 7). In contrast, the injection of caPP2B into *Untrained* cells reduced spike frequencies over the course of 30 LSs, which was very similar to the decreases observed during *in vitro* extinction for *Paired* cells (without caPP2B).

caPP2B injection had no appreciable effect on spike frequency during LSs 1–2 for either *Untrained* (*Untrained + PP2B*; *n* = 11; 6.04 ± 0.33 Hz) or *Paired* cells (*Paired + PP2B*; *n* = 7; 7.50 ± 0.28 Hz). PP2B buffer (*Buffer* control; *n* = 6; 5.83 ± 0.35 Hz) also had no effect on spike frequency during LSs 1–2. During LSs 16–30, *Paired + PP2B* cells showed spike frequencies that were significantly elevated compared to *Paired* (no drug) cells [*F*_(1, 17)_ = 16.71, *p* = 0.001], and slightly (non-significantly) above *Buffer* control levels [*F*_(1, 11)_ = 4.37, *p* = 0.06] (Figure 7B). When measured as a percent change in spike frequencies from the 5th to 30th LS, *Paired + PP2B* cells exhibited a 8.7 ± 4.1% decrease in spiking that was significantly less than *Paired* (no drug) cells (33.5 ± 6.5% decrease) [*F*_(1, 17)_ = 8.11, *p* = 0.011], and no different from *Buffer* cells (2.6 ± 6.0% increase) [*F*_(1, 11)_ = 0.46, *p* = 0.514] (Figure 7C).

In contrast, *Untrained + PP2B* cells showed a progressive reduction in spike frequencies throughout LSs 16–30 that was no different from the decreases observed in *Paired* (no drug) cells [*F*_(1, 21)_ = 2.71, *p* = 0.114], but was significantly greater than *Untrained* [*F*_(1, 24)_ = 20.03, *p* = 0.000] and *Buffer* [*F*_(1, 15)_ = 14.75, *p* = 0.002] cells (Figure 7B). Analysis of the percent change in spike frequencies from the 5th to 30th LS yielded similar results. *Untrained + PP2B* cells showed a large 33.6 ± 3.6% reduction in spiking that was no different than *Paired* (no drug) cells (33.5 ± 6.5% decrease) [*F*_(1, 21)_ = 0.89, *p* = 0.358] (Figure 7C).

In summary, these results partially support the hypothesis that PP2B participates in the reduction in spike frequencies produced by *in vitro* extinction. caPP2B injection into *Untrained* cells decreased spike frequencies, mimicking the effects of *in vitro* extinction. Unexpectedly, injection of caPP2B into *Paired* cells did *not* decrease spike frequencies and blocked the spiking reductions produced by *in vitro* extinction.

### Calyculin a prevents the decrease in spike frequency produced by protein phosphatase 2B in *Untrained* cells

**Figure 8:**
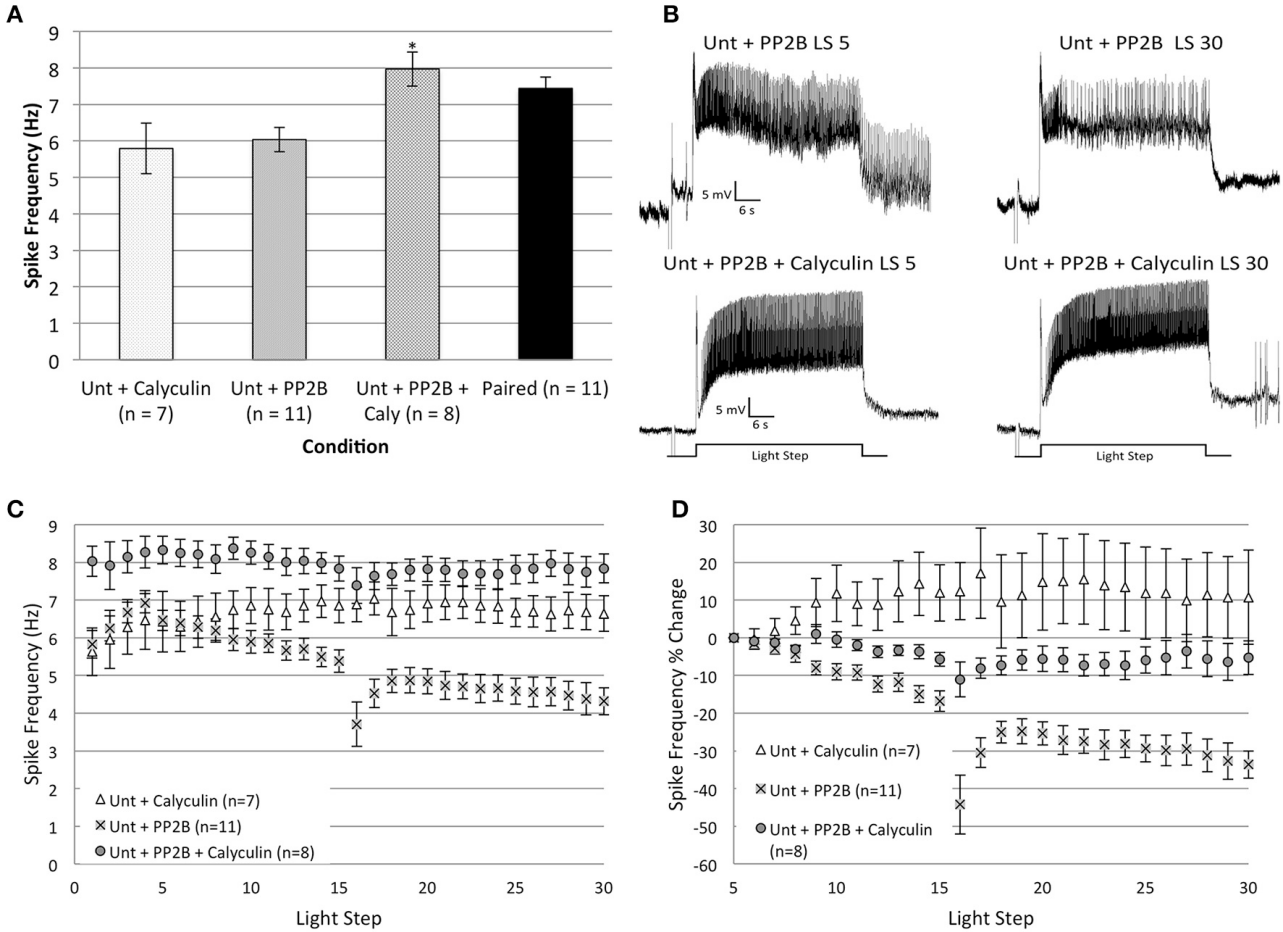
**The incubation of *Untrained* Type B cells in calyculin A (20 nM final bath concentration) for 15 min prevented the reduction in spike frequency produced by injection of catalytically active PP2B (caPP2B) and increased spiking during the first two LSs. (A)** Summary data for average light-evoked spike frequencies during the first two LSs. The combination of caPP2B and calyculin in *Untrained* cells produced higher spike frequencies compared to cells exposed to either caPP2B or calyculin alone. **(B)** Representative light responses recorded from an *Untrained* B cell injected with caPP2B (*Unt + PP2B* group; top trace) and an *Untrained* cell injected with caPP2B in the presence of calyculin (*Unt + PP2B + Calyculin*; bottom trace). The *Unt + PP2B* cell showed a large reduction in spike frequencies over the course of 30 LSs. Calyculin blocked this caPP2B-produced reduction in spike frequency. **(C)** Summary spike frequency data for Type B cells exposed to caPP2B and calyculin across 30 LSs. *Unt + PP2B* cells showed significantly lower spike frequencies during LSs 16–30 compared to *Unt + Calyculin* cells. In contrast, *Unt + PP2B + Calyculin* cells showed a slight increase (non-significant) in spike frequencies compared to *Unt + Calyculin* cells, and were significantly elevated relative to *Unt + PP2B* cells. **(D)** Summary data of the relative percent change in B cell spike frequencies from the 5th to 30th LS. *Unt + PP2B* cells exhibited significant decreases in spike frequency from the 5th to 30th LS (~34%), while *Unt + PP2B + Calyculin* cells showed very little decrease (~5%) over the course of 30 LSs. Note that data for *Unt + Calyculin* and *Unt + PP2B* cells shown here have been replotted from Figures 4, 7. Error bars are ± s.e.m. and significant *p*-values (*p*'s < 0.05) are denoted by an asterisk.

The progressive reduction of spike frequencies produced by *in vitro* extinction in *Paired* cells appears to depend upon both PP2B and PP1, because inhibitors of either phosphatase blocked the spiking reductions. To further probe possible interactions between the two phosphatases, we used calyculin A to block endogenous PP1 activity and injected caPP2B into cells from *Untrained* animals prior to *in vitro* extinction. If the reduction of spike frequency produced by caPP2B occurs through the activation of PP1, then prior incubation in calyculin should block this spiking decrease. This is what we observed (Figures 8B–D).

The combination of caPP2B and calyculin appeared to produce a general facilitatory effect on spike frequencies. An ANOVA of LSs 1–2 showed a significant main effect of treatment condition for *Untrained + PP2B + Calyculin* (*n* = 8), *Untrained + Calyculin* (*n* = 7), *Untrained + PP2B* (*n* = 11), and *Paired* (*n* = 11) groups [*F*_(3, 33)_ = 5.74, *p* = 0.003] (Figure 8A). *Post-hoc* analysis revealed that spike frequencies of *Untrained + PP2B + Calyculin* cells (7.97 ± 0.47 Hz) were significantly greater than *Untrained + PP2B* cells (6.04 ± 0.33 Hz) (*p* = 0.018) and *Untrained + Calyculin* cells (5.79 ± 0.69 Hz) (*p* = 0.016), but were not different from *Paired* (no drug) cells (7.44 ± 0.31 Hz) (*p* = 1.0) (Figure 8A). These data indicated that *Untrained + PP2B + Calyculin* cells enhanced excitability during LSs 1–2 to levels characteristic of *Paired* cells.

During LSs 16–30, calyculin blocked the reduction in spike frequencies produced by caPP2B injection, and spiking of *Untrained + PP2B + Calyculin* cells was not different than *Untrained + Calyculin* cells [*F*_(1, 13)_ = 3.28, *p* = 0.093], but was significantly greater than *Untrained + PP2B* cells [*F*_(1, 17)_ = 40.27, *p* = 0.000] (Figures 8B,C). This block of caPP2B's effects was not a result of general increases in spiking, as similar results were found when spike frequencies were analyzed as a relative percent change from the 5th to 30th LS (Figure 8D). *Untrained + PP2B + Calyculin* cells showed a 5.3 ± 4.6% decrease in spike frequency by the 30th LS that was not significantly different from *Untrained + Calyculin* cells (10.8 ± 12.5% increase) [*F*_(1, 13)_ = 3.35, *p* = 0.090], but was significantly different from *Untrained + PP2B* cells (33.6 ± 3.6% decrease) [*F*_(1, 17)_ = 23.61, *p* = 0.000] (Figure 8D).

In summary, caPP2B did not reduce B cell spike frequencies when endogenous PP1 was inhibited. These results support the view that the reduction in *Untrained* cell spike frequencies produced by caPP2B injection was mediated (at least partly) through endogenous PP1. The combination of calyculin and caPP2B also enhanced spike frequencies of *Untrained* cells during LSs 1–2 to levels comparable to *Paired* cells, suggesting that that caPP2B may have both excitatory and inhibitory effects on B cell spike frequency. When the latter effects were blocked by calyculin, the excitatory effects were unmasked. Our results indicate that the PP1 and PP2B pathways interact to reduce spike frequencies in *Untrained* cells to levels observed during *in vitro* extinction in *Paired* cells.

### Arachidonic acid occludes additional spike frequency decreases produced by extinction in *Paired* cells and mimics the effects of *in vitro* extinction in *Untrained* cells

**Figure 9:**
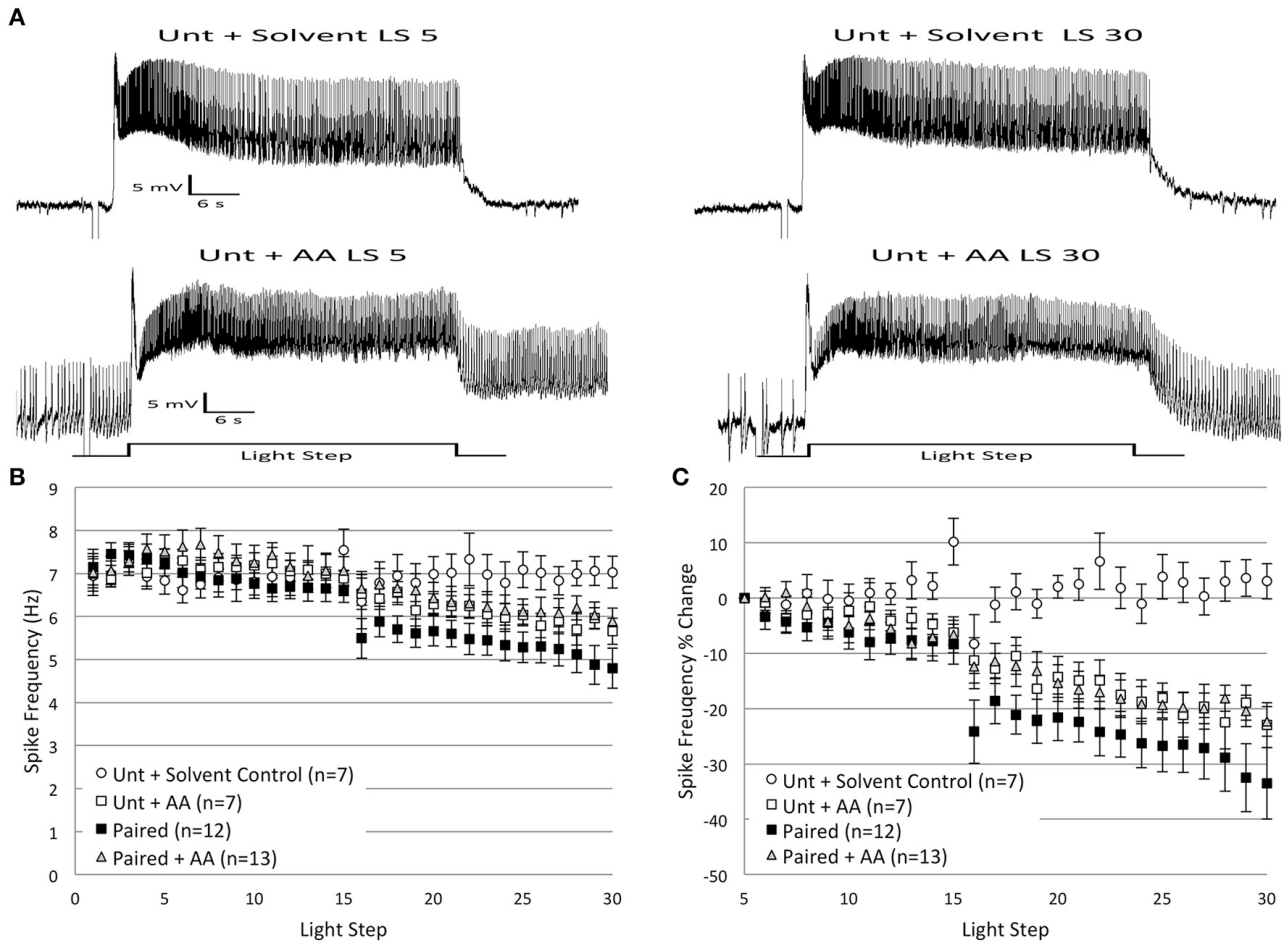
**Bath application of arachidonic acid (AA; 100 μM) onto *Paired* Type B cells reduced B cell spike frequency and occluded any further decreases produced by *in vitro* extinction**. AA applied to *Untrained* B cells produced reductions in spike frequency that mimicked the decreases observed in *Paired* (no drug) cells. AA or vehicle solvent (ethanol, 0.1% final bath concentration) was bath applied to exposed *H.c.* nervous systems immediately after the 5th LS. **(A)** Representative light responses recorded from an *Untrained* Type B cell exposed to solvent (*Unt + Solvent*; top trace) or AA (*Unt + AA*; bottom trace). **(B)** Summary spike frequency data of Type B cells exposed to AA or solvent over 30 LSs. *Paired + AA* cells exhibited a decrease in spike frequency compared to *Unt + Solvent* control cells (significant interaction between LS and condition). The significant reduction in spike frequencies produced in *Paired + AA* cells was as large, but no larger, than the decrease produced in *Paired* (no drug) cells, which suggests that AA occluded the spike frequency reducing effects of repeated LSs in *Paired* (no drug) cells. The application of AA to *Untrained* cells mimicked the effects of *in vitro* extinction and produced reductions in spike frequency that were significantly below levels observed in *Unt + Solvent* control cells. **(C)** Summary data of the relative percent change in B cell spike frequency from the 5th to 30th LS. *Paired + AA* B cells exhibited a significant decline (~22%) in spike frequency over the course of 30 LSs, which was significantly larger than the small changes observed in *Unt + Solvent* control cells (3% increase), but not statistically different from the decreases seen in *Paired* (no drug) cells (~34%). *Unt + AA* cells also showed significantly larger declines in spike frequency (~23%) compared to *Unt + Solvent* controls, and these decreases were not statistically different from *Paired* (no drug) cells. Error bars are ± s.e.m. and significant *p*-values are < 0.05.

Previous data from our lab indicates that AA and one of its principle metabolites, 12(S)-HPETE, contribute to the decrease in Type B cell excitability produced by inhibitory conditioning (Walker et al., 2010). Because AA and 12(S)-HPETE both elicit decreases in B cell light-evoked spike frequencies (similar to the effect of *in vitro* extinction), we hypothesized that these molecules might also be recruited during *in vitro* extinction. To test this, we bath applied AA onto *Untrained* and *Paired* cells during administration of *in vitro* extinction. AA was added to the bath solution after the 5th LS, once spike frequencies had stabilized (as in Walker et al., 2010). If AA is involved in the spike frequency reduction observed during *in vitro* extinction, then the decreases in spiking produced by exogenous AA should occlude further decreases in spike frequencies by repeated LSs in *Paired* cells. In support of this idea, the addition of AA to *Paired* cells produced a reduction in spike frequency that was no larger than the decreases observed in *Paired* (no drug) cells (Figure 9).

Because AA was added after LS 5, an analysis of AA's effects on LSs 1–2 was precluded. During LSs 16–30, application of AA to *Paired* cells (*Paired + AA*; *n* = 13) produced a reduction in spike frequencies that was below *Solvent* control cell (*n* = 7) levels, and became larger over time [significant interaction between LS and condition, corrected for sphericity: *F*_(2.86, 51.42)_ = 5.78, *p* = 0.002] (Figure 9B). The reduction in spike frequency observed in *Paired + AA* cells was slightly (non-significant) less than the reductions produced in *Paired* (no drug) cells [*F*_(1, 23)_ = 3.55, *p* = 0.072] (Figure 9B). Analysis of the percent change in spike frequencies from the 5th to 30th LS further supported these conclusions. *Paired + AA* cells exhibited a 22.3 ± 2.7% decrease in spike frequencies that was significantly greater than *Solvent* control cells (3.0 ± 3.2% increase) [*F*_(1, 18)_ = 11.70, *p* = 0.003], but not significantly different from *Paired* (no drug) cells (33.5 ± 6.5% decrease) [*F*_(1, 23)_ = 2.07, *p* = 0.163] (Figure 9C).

As expected (Walker et al., 2010), the addition of AA to *Untrained* cells (*Untrained + AA*; *n* = 7) produced a decrease in spike frequency that mimicked the reductions produced by *in vitro* extinction. Compared to *Solvent* controls, *Untrained + AA* cells showed progressive reductions in spike frequencies during LSs 16–30 [significant interaction between LS and condition: *F*_(14, 168)_ = 3.72, *p* = 0.000] (Figures 9A,B). The reduced spike frequencies observed in *Untrained + AA* cells were indistinguishable from *Paired* (no drug) cells [*F*_(1, 17)_ = 2.02, *p* = 0.174] (Figure 9B). The effects of AA were more pronounced when considered as a percent change in spiking from the 5th to 30th LS. *Untrained + AA* cells exhibited a 23.0 ± 4.1% decrease in spike frequency by the 30th LS, which was significantly greater than *Solvent* control cells (3.0 ± 3.2% increase) [*F*_(1, 12)_ = 14.25, *p* = 0.003], and not significantly different from *Paired* (no drug) cells (33.5 ± 6.5% decrease) [*F*_(1, 17)_ = 1.86, *p* = 0.191] (Figure 9C).

These findings suggest that AA might be one molecule responsible for the *in vitro* extinction-produced decreases in spike frequency. Not only did AA addition to *Untrained* cells mimic the effects of *in vitro* extinction, but the addition of AA to *Paired* cells also occluded any further declines in spike frequency produced by *in vitro* extinction, suggesting that they are part of a common signaling pathway.

### Baicalein, an inhibitor of 12(S)-hpete production, blocks *in vitro* extinction

**Figure 10:**
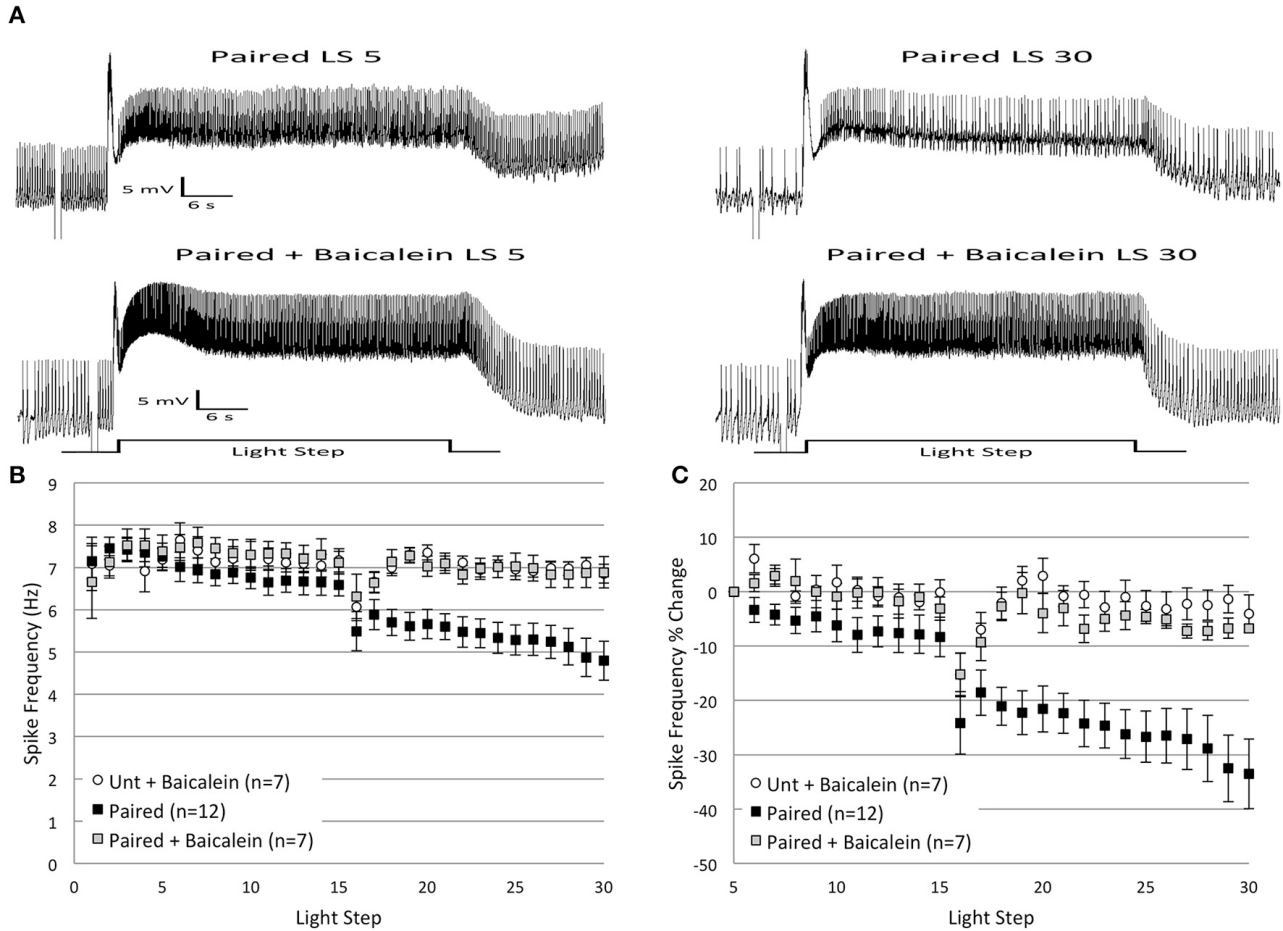
**Incubation of *Paired* Type B cells in baicalein (100 μ M final bath concentration) for 10 min prior to *in vitro* extinction blocked the reduction in spike frequency produced by repeated LSs in *Paired* (no drug) cells. (A)** Representative light responses recorded from a *Paired* Type B cell (top trace) and a *Paired* cell incubated in baicalein (*Paired + Baicalein*; bottom trace). The *Paired* cell showed a clear decline in spike frequency from the 5th to 30th LS, while the *Paired + Baicalein* cell did not. **(B)** Summary spike frequency data of Type B cells exposed to baicalein. The significant decrease in spike frequency seen in *Paired* (no drug) cells during LSs 16–30 was prevented by prior incubation in baicalein. Spike frequencies of *Paired + Baicalein* cells were no different than *Untrained* cells incubated in baicalein (*Unt + Baicalein*). **(C)** Summary data of the percent change in B cell spike frequency from the 5th to 30th LS. The incubation of *Paired* cells in baicalein blocked the large decrease in spike frequency over the course of 30 LSs observed in *Paired* (no drug) cells, and *Paired + Baicalein* cells spiked at frequencies not statistically different from *Unt + Baicalein* cells. Error bars are ± s.e.m. and significant *p*-values are < 0.05.

Our previous research (Walker et al., 2010) found that AA-produced reductions in B cell spike frequency were mainly a result of 12(S)-HPETE, an AA metabolite produced by the 12-lipoxygenase (12-LOX) enzyme. We therefore hypothesized that 12(S)-HPETE might also be a critical metabolite for *in vitro* extinction. We predicted that baicalein, a selective inhibitor of the 12-LOX enzyme, would block the decrease in spike frequency produced by *in vitro* extinction. To test this idea, we incubated *Untrained* and *Paired* cells in baicalein for 10 min prior to the administration of *in vitro* extinction. Consistent with our hypothesis, baicalein significantly blunted the decreases in B cell spike frequency observed in *Paired* cells, but had little impact on *Untrained* cells (Figure 10).

Incubation of *Untrained* and *Paired* cells in baicalein (*Untrained + Baicalein*, *n* = 7, 7.06 ± 0.36 Hz; *Paired + Baicalein*, *n* = 7, 6.89 ± 0.61 Hz) did not alter spike frequencies during LSs 1–2. In contrast, baicalein had a profound effect during LSs 16–30, but only for *Paired* cells. *Paired + Baicalein* cells failed to show the progressive decline in spike frequency during LSs 16–30 and spiked at rates significantly greater than *Paired* (no drug) cells [*F*_(1, 17)_ = 9.17, *p* = 0.008] (Figure 10A), but were no different than *Untrained* [*F*_(1, 20)_ = 0.46, *p* = 0.504] or *Untrained + Baicalein* cells [*F*_(1, 12)_ = 0.02, *p* = 0.897] (Figure 10B). This blockade of the reduction in spike frequency was not a result of non-specific increases in spiking, as spike levels of *Untrained + Baicalein* cells during LSs 16–30 were not different from levels exhibited by *Untrained* (no drug) cells [*F*_(1, 20)_ = 0.65, *p* = 0.428]. Similar conclusions concerning the effects of baicalein were reached when considered as a percent change in spike frequencies from the 5th to 30th LS. *Paired + Baicalein* cells showed a 6.8 ± 0.7% reduction in spike frequency, which was significantly less than *Paired* (no drug) cells (33.5 ± 6.5% decrease) [*F*_(1, 17)_ = 10.46, *p* = 0.005], yet no different than *Untrained* (no drug) cells (2.6 ± 6.0% decrease) [*F*_(1, 20)_ = 0.09, *p* = 0.772], nor from *Untrained + Baicalein* cells (4.1 ± 3.5% decrease) [*F*_(1, 12)_ = 0.79, *p* = 0.393] (Figure 10C).

In summary, baicalein blocked the reduction in spike frequency produced by repeated LSs in *Paired* cells, yet had no effect in *Untrained* cells. These findings support the hypothesis that 12(S)-HPETE is involved in mediating the reduction of B cell spike frequency observed during *in vitro* extinction.

### Calyculin does not block the effects of arachidonic acid

**Figure 11:**
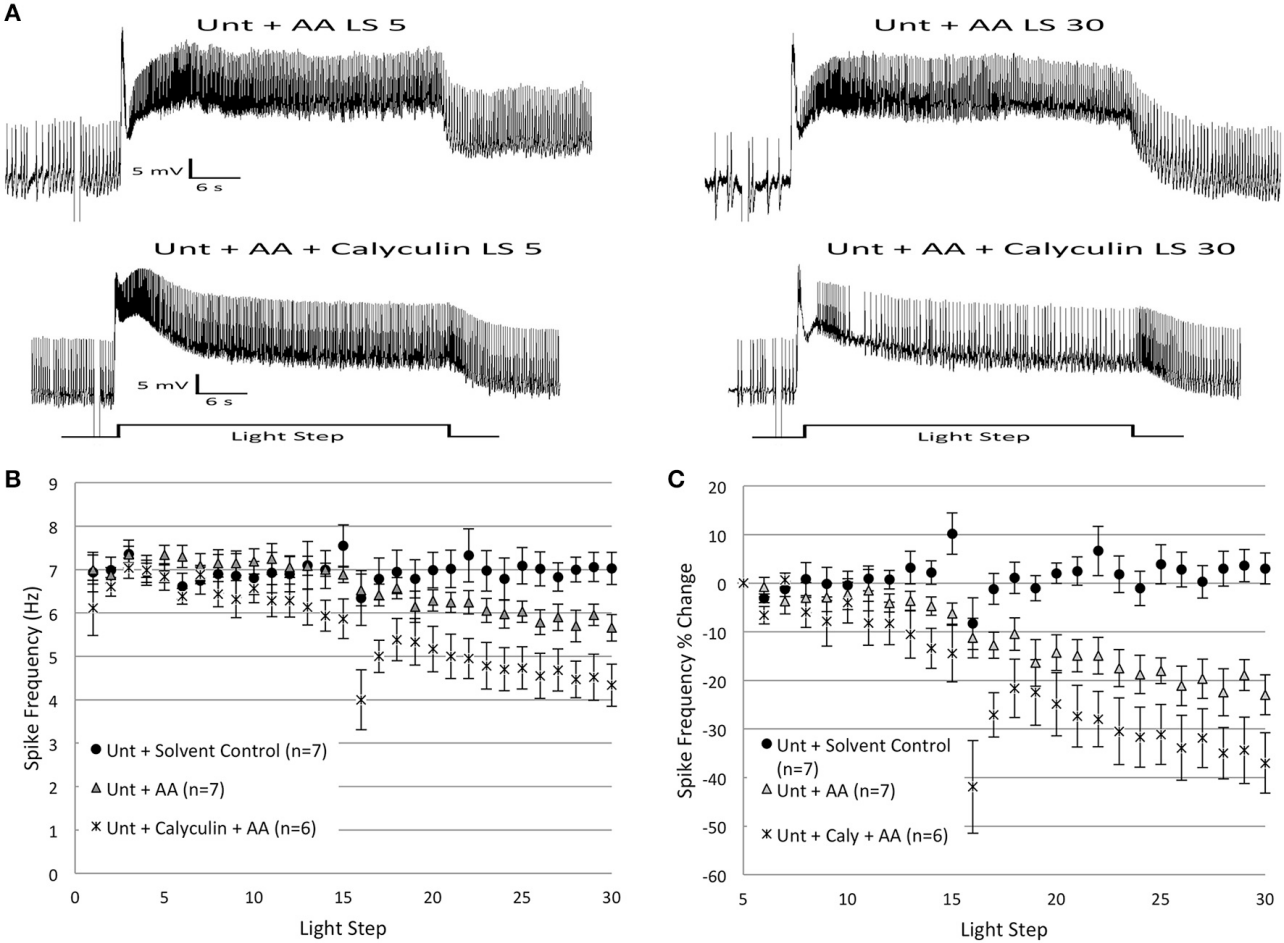
**Incubation of *Untrained* B cells in calyculin A (20 nM final bath concentration) enhanced the reduction in spike frequencies produced by arachidonic acid (AA; 100 μ M final bath concentration). (A)** Representative light responses recorded from an *Untrained* Type B cell exposed to AA (*Unt + AA*; top trace) or calyculin followed by AA (*Unt + Calyculin + AA*; bottom trace). **(B)** Summary spike frequency data over 30 LSs of Type B cells exposed to AA or calyculin. *Unt + AA* cells exhibited decreases in spike frequencies compared to *Unt + Solvent* control cells (significant interaction between LS and condition). *Unt + Calyculin + AA* cells showed decreases in spike frequencies that were significantly larger than the decreases observed in *Unt + AA* cells. **(C)** Summary data of the relative percent change in B cell spike frequency from the 5th to 30th LS. *Unt + Calyculin + AA* cells showed a large 37% decline in spike frequency that was significantly greater than the ~3% increase seen in *Unt + Solvent* control cells, and qualitatively (though not statistically) larger than the ~23% reduction observed in *Unt + AA* cells. Error bars are ± s.e.m. and significant *p*-values are < 0.05.

Precedents exist for an interaction between AA/lipoxygenase metabolites, protein phosphatases, and K^+^ channels (e.g., Duerson et al., 1996). Because the addition of exogenous caPP1 and AA both separately reduced B cell spike frequencies over the course of 30 LSs in *Untrained* cells, we wondered if they did so by participation in the same, or different, signaling pathway(s). To investigate these possibilities, we exposed B cells to AA in the presence of the PP1 inhibitor, calyculin A. B cells were first incubated in calyculin for 15 min, followed by the bath application of AA after the 5th LS. If the spike frequency decreases produced by AA are mediated through a PP1-dependent pathway, then incubation of B cells in calyculin prior to AA addition should prevent/attenuate the reduction in spike frequency. Our results failed to support this view. We found instead that calyculin did not block the reductions in spike frequency produced by AA and actually led to greater reductions in spiking (Figure 11) than AA-alone.

*Untrained* cells incubated in calyculin and exposed to AA (*Untrained + Calyculin + AA; n* = 6) showed marked reductions in spike frequencies during LSs 16–30 that were significantly below levels observed in *Untrained + Calyculin* [*F*_(1, 11)_ = 10.14, *p* = 0.009], and AA vehicle *Solvent* control cells [*F*_(1, 11)_ = 11.69, *p* = 0.006] (Figure 11B). This reduction was significantly greater than that observed in *Untrained + AA* cells [*F*_(1, 11)_ = 6.75, *p* = 0.025] (Figures 11A,B), and was no different than the reductions observed in *Paired* (no drug) cells [*F*_(1, 16)_ = 1.06, *p* = 0.318].

Similar effects were observed when spike frequencies were analyzed as a percent change in spiking from the 5th to 30th LS. *Untrained + Calyculin + AA* cells exhibited a 37.0 ± 6.2% decrease in spike frequency that was significantly greater than *Untrained + Calyculin* cells (10.8 ± 12.5% increase) [*F*_(1, 11)_ = 9.39, *p* = 0.011] (Figure 11C), and *Solvent* controls (3.0 ± 3.2% increase) [*F*_(1, 11)_ = 20.60, *p* = 0.001]. The 37.0 ± 6.2% decrease in spike frequency observed in *Untrained + Calyculin + AA* cells was qualitatively, though not statistically, greater than *Untrained + AA* cells (23.0 ± 4.1% decrease) [*F*_(1, 11)_ = 3.82, *p* = 0.077], and was not significantly different from *Paired* (no drug) cells (33.5 ± 6.5% reduction) [*F*_(1, 16)_ = 0.46, *p* = 0.508].

In summary, calyculin failed to block the reduction in spike frequencies produced by AA, and instead enhanced the inhibitory effect of AA on spike frequency. These results indicate that the effects of AA are not mediated by PP1, and it seems unlikely that PP1 lies downstream from AA in B cell signaling pathways. However, it is possible that the reverse is true: activation of PP1 (by LSs and PP2B in *Paired* cells) might be critical for synthesis/release of AA (and/or activity of 12-LOX). Our results suggest that there may also be an inhibitory interaction between the PP1- and AA-signaling pathways in B cells, because the combination of calyculin and AA led to greater decreases in spike frequencies than AA alone.

### Delayed *in vitro* extinction fails to produce reductions in spike frequency

Prior research has demonstrated that the interval between the end of learning acquisition and the beginning of extinction training can affect the outcome of extinction (Maren and Chang, 2006; Myers et al., 2006; Cavallo et al., 2014). Extinction training conducted shortly after associative conditioning can improve the effectiveness of extinction, and may even abolish the original associative memory (Mao et al., 2006; Myers et al., 2006; Monfils et al., 2009). In other circumstances, delayed extinction is more effective at producing lasting CR reduction without detectable evidence of the original memory (i.e., no spontaneous recovery) (Maren and Chang, 2006).

To identify whether similar differences existed for extinction effectiveness in *H.c.*, we repeated the *in vitro* extinction experiments using a prolonged (24 h) interval between original acquisition (light-rotation pairings) and the beginning of *in vitro* extinction. We hypothesized that memory consolidation processes would strengthen the memory for paired conditioning during the 24 h period after conditioning (Crow and Forrester, 1990; Epstein et al., 2003). Consequently, we expected that delayed *in vitro* extinction would produce smaller reductions in B cell spike frequencies (our cellular correlate of *H.c.* extinction) compared to more immediate (1–2 h) *in vitro* extinction. Thus, animals were given 2 days of paired conditioning followed by a 24 h interval (*Paired 24-h* group), before receiving *in vitro* extinction (two sets of 15 LSs), using the same methodology as reported above. This longer interval is in contrast to the *Paired* group discussed above, which received *in vitro* extinction 1–2 h after the end of paired conditioning (referred to hereafter as the *Paired 2-h* group).

**Figure 12:**
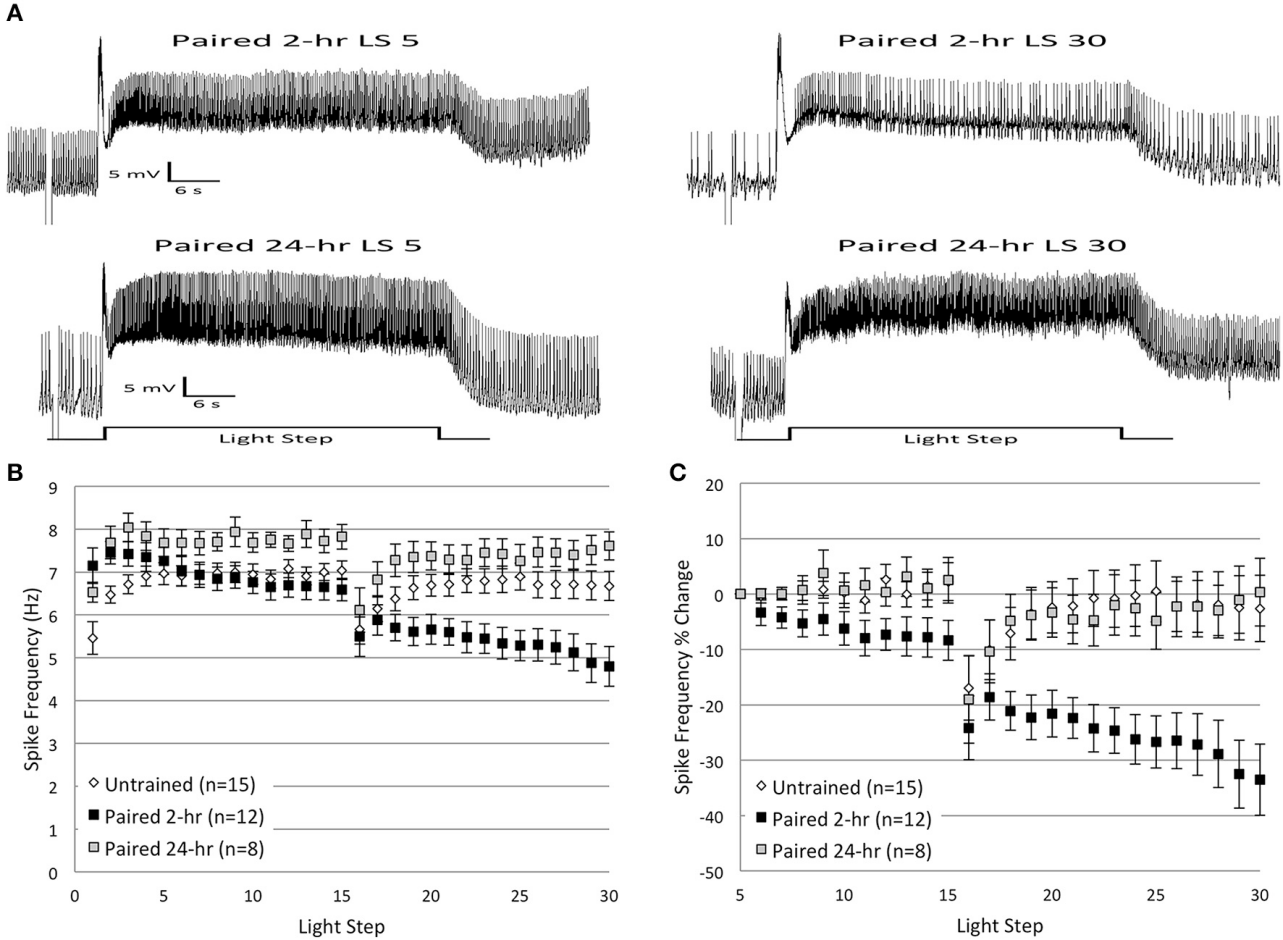
**Delayed *in vitro* extinction failed to reduce Type B cell spike frequencies. (A)** Representative light responses recorded from Type B photoreceptors from paired-trained animals either 2 h (*Paired 2-h*; top trace) or 24 h (*Paired 24-h*; bottom trace) after the end of paired conditioning. The *Paired 24-h* cell did not show any appreciable decline in spike frequency over the course of 30 LSs, while the *Paired 2-h* cell showed a significant decline. **(B)** Summary spike frequency data of Type B cells across 30 LSs from *Untrained* or *Paired* animals, both 2 and 24 h after conditioning. *Paired 24-h* cells showed increased spike frequencies compared to *Untrained* control and *Paired 2-h* cells during LSs 1–30, and failed to show the progressive decrease in spike frequency exhibited by *Paired 2-h* cells. **(C)** Summary data of the percent change in B cell spike frequency from the 5th to 30th LS. *Paired 24-h* cells showed no change (~0%) in spike frequency over the course of 30 LSs, which was indistinguishable from control cells (~3% decrease), yet significantly different from the ~34% spike frequency decrease exhibited by *Paired 2-h* cells. Error bars are ± s.e.m. and significant *p*-values are < 0.05. Note that one cell from each of the *Paired 2-h and Paired 24-h* groups was excluded from analysis during LSs 1–2 due to missing spike frequency data as a result of electrode plugging.

During LSs 1–2, *Paired 24-h* cells exhibited spike frequencies similar to that of *Paired 2-h* cells. Thereafter, however, *Paired 24-h* cells failed to exhibit the characteristic decrease in spiking observed in *Paired 2-h* cells (Figures 12A–C) and had higher levels of spike frequencies during LSs 23–30 than *Paired 2-h* cells. These results indicate that *in vitro* extinction was ineffective at altering B cell excitability when delayed by 24 h.

During LSs 1-2, spike frequencies of *Paired 24-h* cells (*n* = 7, 7.23 ± 0.27 Hz) were greater than *Untrained* (*n* = 16, 5.96 ± 0.26 Hz) control cells, and comparable to *Paired 2-h* cells (*n* = 11, 7.44 ± 0.31; see Figure 2B), indicative of the enhanced excitability produced by associative learning (Farley and Alkon, 1982, 1987). An ANOVA compared the average spike frequencies during LSs 1–2 for *Paired 24-h*, *Paired 2-h*, and *Untrained* cells, and found a significant main effect of treatment condition [*F*_(2, 31)_ = 8.66, *p* = 0.001]. *Post-hoc* analysis revealed that both *Paired 24-h* and *Paired 2-h* cells spiked significantly more frequently than *Untrained* cells (*p*'s = 0.023, 0.002, respectively), but did not differ from each other (*p* = 1.0). After the first few LSs, spike frequencies of *Paired 24-h* cells were relatively constant and did not diminish with successive LSs, unlike *Paired 2-h* cells (Figures 12A–C). *Paired 24-h* cells showed enhanced rates of spiking throughout LSs 1–30 that were significantly greater than *Untrained* cells [*F*_(1, 21)_ = 5.38, *p* = 0.030] (Figure 12B). *Paired 24-h* (*n* = 8) cells also showed significantly greater spike frequencies than *Paired 2-h* (*n* = 12) cells over LSs 1–30 [*F*_(1, 18)_ = 10.45, *p* = 0.005], and failed to show the progressive decline in spike frequencies observed in *Paired 2-h* cells as a result of repetitive LSs (Figure 12B).

Similar results were found when spike frequencies were analyzed as a percent change from the 5th to 30th LS. *Paired 24-h* cells showed little change in spike frequency during LSs 5–30 (0.4 ± 6.1% decrease), which was significantly different from *Paired 2-h* cells (33.5 ± 6.5% decrease) [*F*_(1, 18)_ = 9.61, *p* = 0.006], but not different from *Untrained* cells (2.6 ± 6.0% decrease) [*F*_(1, 21)_ = 0.00, *p* = 0.968] (Figure 12C).

These results indicate that exposure of B cells to repeated LSs produced a large decrease in spike frequency when given to *Paired* cells relatively soon (1–2 h) after conditioning. Delaying this presentation by 24 h abolished the decrease in spike frequency produced by *in vitro* extinction.

## Discussion

### Cellular and molecular correlates of extinction training

The use of an *in vitro* extinction protocol allowed us to simulate whole-animal extinction in isolated *H.c.* nervous systems and investigate the cellular and molecular processes that occur during extinction training. Type B cells from paired-trained animals exposed to thirty successive LSs showed large (33.5%) and progressive reductions in spike frequencies that were significantly below *Untrained* and non-associative control (*Random*) levels. These spike frequency reductions provided a striking correlate of whole-animal behavioral extinction (Cavallo et al., 2014), because they were contingent upon prior associative conditioning and only occurred when *in vitro* extinction was administered 1–2 h, but not 24 h, after paired conditioning. Our results implicated three molecules as participating in mediating the effects of *in vitro* extinction: PP1, PP2B, and AA/12(S)-HPETE.

#### Protein phosphatase 1

Three sets of experiments suggest that PP1 participates in the decline in spike frequency produced by *in vitro* extinction. First, incubation of B cells in the PP1 inhibitor, calyculin A, blocked the progressive spiking decrease in *Paired* cells, but had no significant effects in *Untrained* cells. We attribute this blocked reduction to PP1 inhibition, because although calyculin also inhibits PP2A (calyculin IC_50_ values of 0.5–1 nM for PP2A vs. 2 nM for PP1), several previous findings argue against any major role of PP2A. Huang and Farley (2001) found that cantharidic acid, a potent PP2A-selective inhibitor, had negligible effects on B cell excitability during *in vitro* associative conditioning, while both calyculin and inhibitor-1 had substantial impacts on cumulative depolarization and suppression of K^+^ currents. Second, the injection of caPP1 into *Untrained* cells reduced spike frequencies by 20%, which was not statistically different from the 33.5% decrease observed in *Paired* cells during *in vitro* extinction. Third, caPP1 injection into *Paired* cells reduced spike frequencies and occluded any further declines by *in vitro* extinction. This suggests that the declines in spiking elicited by repetitive LSs utilize PP1 signaling.

At least two alternative interpretations regarding the caPP1 results are possible. First, the spiking reductions during LSs 16–30 for *Paired + PP1* cells might have reflected slow-to-develop effects of the enzyme itself, rather than effects triggered by LSs in *Paired* cells. From this perspective, exogenous caPP1 reduced spiking and occluded further reductions caused by repeated LSs. By this hypothesis, the latter effects arise from LS-induced activation of endogenous PP1. A second account views the reductions in spiking for *Paired + PP1* cells resulting from effects of the LSs (just as for *Paired* cells), which then further occluded additional reductions due to injections of exogenous caPP1 (as occurred for *Untrained + PP1*). In both accounts, an occlusion between the effects of exogenous caPP1 and processes triggered by LSs is occurring, but it is unclear which is occluding which. Despite this uncertainty, the main point is clear. The reductions in spiking produced by caPP1 (in *Untrained* cells) and by LS-triggered processes (in *Paired* cells) are occurring in a common pathway in the case of *Paired + PP1* cells. When combined, these reductions fail to add.

**Figure 13:**
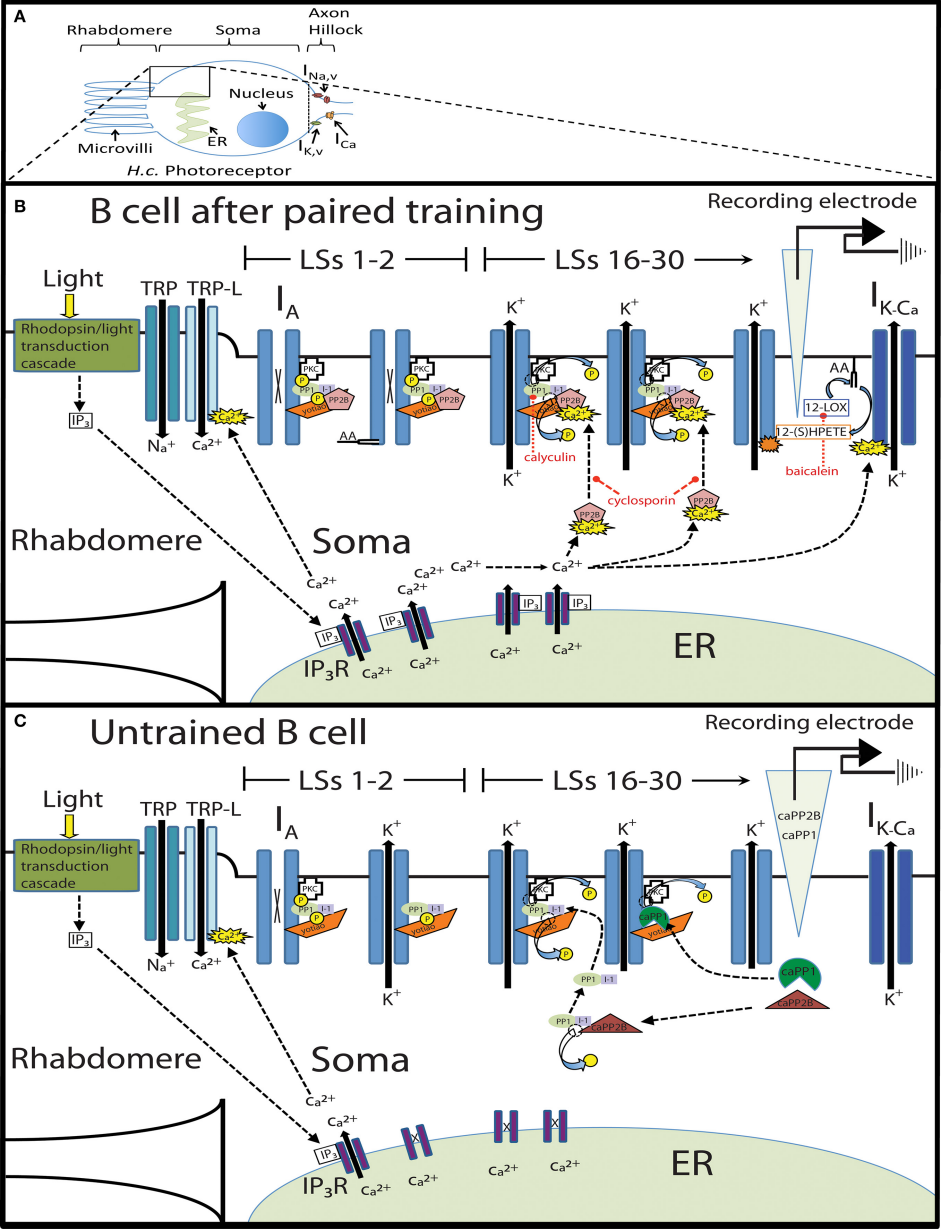
**Molecular model of a *Paired* and *Untrained* Type B cell given repeated light steps. (A)** A cartoon illustration of a single Type B photoreceptor with expanded view of the rhabdomere and soma subcellular regions. Note the three classes of voltage-dependent ion channels (sodium, potassium, and calcium) located in the axon hillock region (indicated by the dotted line). **(B)** Expanded view of a Type B cell 1–2 h after paired training. During light steps (LSs) 1–2 (left), many (~30–50%) of the somatic somatic I_A_ K^+^ channels are shut (indicated by an X in the channel pore) due to PKC-mediated phosphorylation of the channel itself or channel-associated proteins (P indicates a phosphate group). Paired conditioning also mobilizes inhibitor-1/PP1 complexes and PP2B to K^+^ channel signaling complexes, where both inactive phosphatases are localized via anchoring proteins such as yotiao/AKAP. Paired conditioning also activates AA, which facilitates the phosphorylation of K^+^ channel signaling complexes. The light-induced phototransduction cascade involving rhodopsin and a still unidentified G-protein coupled receptor (GPCR), results in the production of inositol trisphosphate (IP_3_). Liberated IP_3_ subsequently activates IP_3_ receptors (IP_3_Rs) and releases Ca^2+^ from the endoplasmic reticulum (ER) into the cytosol. Paired conditioning leads to larger than normal light-induced Ca^2+^ release from the ER, and each successive LS (i.e., LSs 16–30) (right) releases a large amount of Ca^2+^ that favors the activation of PP2B. Activated PP2B disinhibits PP1 (through the dephosphorylation of inhibitor-1, I-1), which is now able to dephosphorylate K^+^ channels (or associated proteins), increase the activity of I_A_ channels, and reduce B cell spike frequency. The spike frequency reducing effects of PP2B and PP1 during the later LSs can be blocked by the inhibitors cyclosporin and calyculin, respectively (indicated by red dashed lines). Concurrent to the phosphatase-signaling pathway, repeated LSs also initiate 12-LOX activity, which metabolizes AA into 12(S)-HPETE. 12(S)-HPETE increases the activity of I_A_ K^+^ channels, and possibly enhances the influx of Ca^2+^ via transient receptor potential-like (TRP-L) channels. Both of these processes further reduce B cell excitability during LSs 16–30. This reduced excitability might be further potentiated by greater activation of I_K-Ca_ via large Ca^2+^ release from the ER. **(C)** Expanded view of an *Untrained* Type B cell during LSs 1–2 (left) and LSs 16–30 (right) injected with either catalytically active PP1 (caPP1) or PP2B (caPP2B). During the first two LSs, a certain fraction of somatic K^+^ channels are phosphorylated via constitutively active PKC or other kinase(s), but most channels are not phosphorylated, resulting in the basal level of B cell excitability (i.e., lower than in *Paired* cells). Injection of caPP1 into *Untrained* B cells leads to the dephosphorylation of a certain fraction of phosphorylated K^+^ channel signaling complexes (or associated proteins), enhancing K^+^ channel activity, which reduces B cell excitability below baseline levels during LSs 16–30. Similarly, injection of caPP2B disinhibits endogenous PP1, which eventually localizes to K^+^ channel signaling complexes (via yotiao) and increases the activity of I_A_ K^+^ channels, and reduces B cell spike frequency. Dotted arrows indicate direction of action, while solid arrows show ionic movement across a membrane through various ion channels.

These results point toward PP1 signaling as being a critical pathway that mediates decreases in B cell excitability produced during *in vitro* extinction. Based on our prior work showing that PP1 enhances the same somatic K^+^ channel currents reduced by associative conditioning (Huang and Farley, 2001), we posit that during *in vitro* extinction, PP1 reverses the cellular effects of paired conditioning through dephosphorylation of K^+^ channels (or associated proteins) that are phosphorylated by PKC (Farley and Auerbach, 1986; Farley and Schuman, 1991) (see Figure 13).

#### Protein phosphatase 2B

Two findings argue for the involvement of PP2B signaling in the decreased excitability produced by *in vitro* extinction. First, incubation of B cells in the PP2B-specific inhibitor, cyclosporin A, prior to *in vitro* extinction completely blocked the large reduction in spiking elicited in *Paired* cells without drug, but did not significantly affect *Untrained* cells. Second, injection of caPP2B into *Untrained* cells elicited a 33.6% decrease in spiking that was essentially identical to the 33.5% decline observed during *in vitro* extinction in *Paired* cells. The caPP2B-produced spiking reduction in *Untrained* cells was dependent on PP1 activity, as it was blocked by the PP1 inhibitor calyculin A. This finding, combined with the fact that calyculin produced a similar block of spike frequency reductions in *Paired* cells, suggests that in *Paired* cells, PP2B activation acts upstream of PP1 to reduce B cell spiking during *in vitro* extinction.

One unexpected finding concerned the failure of caPP2B to reduce spiking when injected into *Paired* cells and to occlude any further declines in spike frequencies produced by repetitive LSs. This failure might be a consequence of injecting untargeted caPP2B into B cells, which might fail to dephosphorylate the substrates normally exposed to PP2B during *in vitro* extinction. We propose that endogenous PP2B is likely targeted to subcellular A-type K^+^-channel signaling complexes, through its associated regulatory subunit (Cohen, 1989), where it acts to disinhibit PP1 (through inactivation of inhibitor-1) and reduce B cell excitability. In contrast, injection of exogenous caPP2B (without a regulatory subunit), might not preferentially associate with these K^+^-channel signaling complexes, and thus would fail to activate PP1. caPP2B might even dephosphorylate other targets that endogenous PP2B would not normally encounter [e.g., voltage-gated Ca^2+^ channels (Chad and Eckert, 1986)], and thereby oppose the reductions in B cell excitability produced by PP2B-mediated disinhibition of PP1.

In summary, our results suggest that PP2B is critically involved in the spike frequency reduction produced by *in vitro* extinction. PP2B may reduce B cell excitability through the disinhibition of PP1, and the subsequent dephosphorylation and opening of somatic K^+^ channels, reversing learning-produced increases in excitability. This finding is reminiscent of work in rats indicating that PP2B mediates the extinction of conditioned fear responses and reverses the molecular processes (e.g., Akt kinase phosphorylation) associated with fear conditioning (Lin et al., 2003).

#### Specificity of phosphatase inhibitors calyculin A and cyclosporin A

A legitimate issue in our use of calyculin and cyclosporin concerns the cross-species similarity of their intended protein phosphatase targets, and whether these inhibitors have similar substrate specificity in *H.c.* neurons. We believe the likelihood that these molecules exhibit similar functionality in *H.c.* is high for two reasons, (1) Biochemical analysis of phosphatases in invertebrate preparations, including starfish (Pondhaven and Cohen, 1987), *Drosophila* (Orgad et al., 1987), and *Aplysia* (Ichinose et al., 1990; Endo et al., 1995), indicate that their properties are remarkably similar to corresponding proteins in mammals (e.g., dephosphorylation of the beta subunit of phosphorylase kinase, IC_50_ values for inhibitors-1 and -2, etc.), and (2) Genomic and crystallographic studies suggest striking conservation of phosphatase structures across species. For instance, genomic analyses indicate that the catalytic subunit of PP1 is one of the most conserved eukaryotic proteins (Ceulemans and Bollen, 2004; Shi, 2009), with ~70% or greater protein sequence identity. Crystallographic studies of PP1 indicate that the active site is comprised of multiple calyculin-interaction regions, which prevent binding to downstream substrates (Lindvall et al., 1997; Kita et al., 2002). Thus, the highly conserved amino acid sequence and structure of PP1's active site make similar pharmacological and functional profiles for calyculin-produced inhibition of PP1 in *H.c.* neurons likely.

Cyclosporin has been used as an inhibitor of PP2B in numerous species (see Rühlmann and Nordheim, 1997 for review), ranging from yeast (Marton et al., 1998) to humans. The mechanism for cyclosporin's inhibition of PP2B has been extensively studied (Huai et al., 2002), and there appears to be strict conservation throughout all eukaryotes, such that all PP2B A genes encode for a polypeptide that distinguishes PP2B from other phosphatases. As with calyculin, it therefore seems unlikely that cyclosporin would have reliable effects in organisms as distant as yeast and humans, but not have noticeable effects in *H.c.* Although molecular identification and biochemical characterization of phosphatases from *H.c.* has not yet been accomplished, it seems highly likely that PP1 and PP2B were the major targets affected by calyculin and cyclosporin, respectively.

#### Arachidonic acid and 12(S)-HPETE

Three lines of evidence point to AA and 12(S)-HPETE as important contributors to the spike frequency decrease produced by *in vitro* extinction. First, baicalein, a specific 12-LOX enzyme inhibitor, blocked the spike frequency reduction observed in *Paired* cells, yet had no effect in *Untrained* cells. The 12-LOX enzyme is one of several intermediary enzymes that metabolize AA into second messenger molecules (Marks and Furstenberger, 1999). Our prior work in *H.c.* (Walker et al., 2010) found 12(S)-HPETE to be a main AA metabolite mediating the B cell spiking decreases produced by both AA application and inhibitory conditioning.

The block of extinction-produced reductions in spike frequency by baicalein was somewhat surprising given the evident involvement of phosphatase-signaling pathways in *H.c.* extinction. Baicalein would not be expected to directly impact the activity of PP1 or PP2B, leaving the phosphatase-signaling pathway capable of activating K^+^ channels and reducing spike frequencies. One possible explanation might be that baicalein indirectly interferes with the PP2B-PP1 pathway through the disruption of intracellular Ca^2+^ signaling that relies on AA/12(S)-HPETE to activate Ca^2+^ entry pathways, such as the transient receptor potential-like (TRP-L and/or TRP) (Chyb et al., 1999) and arachidonate-regulated Ca^2+^ (ARC) channels (Shuttleworth et al., 2004; Meves, 2008). Although the precise mechanism of this AA/12(S)-HPETE/phosphatase relationship is unclear, our novel finding that calyculin enhanced the reduction of spiking produced by AA in *Untrained* B cells supports the idea that both signaling pathways interact in a restrictive way.

The second finding that implicates AA/12(S)-HPETE involvement during *in vitro* extinction is that application of AA to *Untrained* cells elicited a progressive 23.0% reduction in spiking that mimicked the effects of *in vitro* extinction in *Paired* cells. This decrease was slightly (though not significantly) less than the 33.5% decrease produced in *Paired* (no drug) cells during *in vitro* extinction, which might indicate that AA/12(S)-HPETE is not the only molecule utilized during *in vitro* extinction, and/or that the pharmacokinetics of AA/12(S)-HPETE production are different for exogenous AA application vs. physiological stimulation. The AA-produced spiking decrease replicates prior research in *H.c.*, which showed that application of AA or 12(S)-HPETE reduced spiking in *Untrained* B cells (Walker et al., 2010). Walker et al. (2010) also found that AA enhanced the transient I_A_ K^+^ current, the same current that is reduced following paired conditioning (Alkon et al., 1985; Farley, 1988a) and increased following inhibitory conditioning (Farley et al., submitted). Based on these findings, AA/12(S)-HPETE-produced increases in I_A_ K^+^ currents are likely one mechanism that contributes to the reduction in B cell spiking during *in vitro* extinction (see Figure 13).

The third result that suggests AA/12(S)-HPETE is utilized during *in vitro* extinction is that the reduction in spike frequency produced by AA application to *Paired* cells occluded any further declines as a result of repeated LSs. This suggests that AA signaling and *in vitro* extinction share a common molecular pathway. Our previous experiments with AA (Walker et al., 2010) suggest that the AA-produced occlusion of additional spike frequency reductions observed here for *Paired + AA* cells is unlikely to represent a “floor” effect in spike frequency reduction. Walker et al. (2010) found that the application of AA to B cells from inhibitory-conditioned animals produced an additional ~22% reduction in spike frequency beyond that elicited by inhibitory conditioning alone. The somewhat smaller spiking reduction observed presently in *Paired + AA* cells compared to *Paired* (no drug) cells might be a result of AA-produced enhancements of PKC activity (Lester et al., 1991), which could temporarily increase B cell excitability. This possibility was raised by Walker et al. (2010) to explain a brief elevation in B cell spike frequency produced by inhibitory conditioning, which dissipated 24 h later. We suggested that although AA metabolites like 12(S)-HPETE might reduce spiking over time, AA itself might transiently activate PKC and temporarily increase, rather than decrease, spiking (Walker et al., 2010). The present application of AA to *Paired* cells could have created a large fraction of non-metabolized AA that partially elevated B cell spiking through its facilitation of PKC and the resulting PKC-mediated reduction of I_A_. Our baicalein results indicate, however, that *in vitro* extinction favors AA metabolism and the activity of 12(S)-HPETE.

### Molecular model of extinction in *H.c.*

Collectively, our results support the conclusion that PP1, PP2B, and AA/12(S)-HPETE mediate reductions in B cell spike frequency produced by *in vitro* extinction. Here we propose a molecular model of extinction that incorporates this evidence into the broader body literature in *H.c*. (Figure 13). As previously proposed (Farley and Auerbach, 1986), paired conditioning activates PKC through the simultaneous release of two PKC cofactors, Ca^2+^ and diacylglycerol (DAG). Ca^2+^ release results primarily from a phototransduction cascade involving rhodopsin, phospholipase C, and the production of inositol trisphosphate (IP_3_) (Sakakibara et al., 1994), which activates IP_3_ receptors and releases Ca^2+^ from the endoplasmic reticulum into the cytosol, a process thought to be critical for learning-produced alterations of K^+^ conductances and B cell excitability (Sakakibara et al., 1986). DAG production results from rotation-induced activation of G-protein coupled GABA_B_ and 5-HT receptors (see Blackwell and Farley, 2009 for review). Activated PKC is mobilized to the plasmamembrane where it reduces the activity of multiple classes of somatic K^+^ channels (e.g., I_A_ and I_K−Ca_), causing enhanced B cell excitability (Farley and Auerbach, 1986; Farley and Schuman, 1991). We suggest that endogenous PP1 is also localized to somatic K^+^ channel signaling complexes, where it remains inactive due to inhibition by inhibitor-1. Rotation given during associative conditioning also stimulates the cleavage of AA from membrane phospholipids (via GABA_B_ activation), which has been hypothesized to augment PKC effects and further enhance B cell excitability (Lester et al., 1991; Talk et al., 1997; Muzzio et al., 2001).

Shortly (1–2 h) after paired conditioning (Figure 13A), *in vitro* extinction (repeated LSs) elicits larger than normal increases in cytosolic Ca^2+^ (Ito et al., 1994). We suggest that elevated intracellular Ca^2+^ preferentially activates PP2B due to PP2B's high affinity for Ca^2+^ compared to PKC (Lisman, 1989), and saturation of PKC signaling. Activated PP2B aggregates near PP1-containing K^+^ channel complexes through regulatory subunit targeting (e.g., yotiao/AKAP), where it dephosphorylates inhibitor-1 and relieves PP1 inhibition (Mulkey et al., 1994). Enzymatically active PP1 is now able to enhance the activity of A-type K^+^ channels and reduce B cell spiking. Exposure to cyclosporin or calyculin after paired conditioning blocks this phosphatase-signaling pathway and prevents *in vitro* extinction from reducing B cell spike frequencies. Concurrent with the PP2B-PP1 signaling pathway, AA is metabolized by the 12-LOX enzyme into 12(S)-HPETE, which further reduces B cell excitability through enhancements of I_A_. The mechanism by which 12-LOX is activated by repeated LSs in *Paired* cells is presently unknown.

Our experiments using catalytically active phosphatases (caPP1 and caPP2B) into *Untrained* B cells are congruent with this model (Figure 13B). Injection of caPP2B into *Untrained* cells might dephosphorylate a large fraction of cytosolic inhibitor-1/PP1 complexes that have not been mobilized to the membrane. Once dephosphorylated, activated PP1 anchors to stabilizing proteins (e.g., yotiao) and dephosphorylates K^+^ channel signaling-complexes (or associated proteins). Injection of caPP1 into *Untrained* B cells has similar, but less robust, effects on K^+^ channel activity. Although caPP1 is able to dephosphorylate a certain percentage of K^+^ channel signaling-complexes (or associated proteins), we suggest that the lack of a regulatory subunit prevents localization to K^+^ channel signaling-complexes, and therefore results in less dephosphorylation.

In contrast to *in vitro* extinction given shortly after associative conditioning, delayed *in vitro* extinction did not decrease B cell spike frequencies. Two possible explanations present themselves. First, 24 h after associative conditioning, repeated LSs might activate PP2B, PP1, and AA/12(S)-HPETE, but their ability to reduce spike frequencies (through enhancements of I_A_), is counteracted and overshadowed by persistent, constitutive PKC activity (Farley and Schuman, 1991), which suppresses I_A_ and increases cell excitability, thus preventing reductions in spike frequencies. Second, perhaps phosphatases are unable to significantly impact B cell spiking during delayed *in vitro* extinction because phosphatase-signaling effects are restricted to a relatively brief critical period after paired conditioning. Although numerous mechanisms could create the conditions for such a critical period, one possibility is that phosphatase activation shortly after paired conditioning disrupts the consolidation of associative memories by regulating the activity of cAMP response element-binding protein (CREB) (Silva et al., 1998), which could inhibit additional synthesis of PKC necessary for producing the behavioral changes indicative of long-term associative memories in *H.c.* (Alkon et al., 2005).

### Relationship to extinction in other systems

Our evidence indicates that PP1, PP2B, and AA/12(S)-HPETE all participate in the *in vitro* extinction-produced reduction of B cell spiking. Therefore, these molecules are likely candidates for mediating the cellular changes in excitability that accompany the extinction-produced reversal of phototactic suppression. Not surprisingly, there are precedents for involvement of these same (or closely-related) signaling pathways in extinction of memories in other systems.

For example, a similar bi-directional modification of K^+^ channels by PKC/PKA and PP1 is believed to underlie short-term facilitation and depression of sensory-motor neuron synaptic transmission in *Aplysia* (Braha et al., 1990; Sugita et al., 1994; Endo et al., 1995). Additionally, in rat hippocampal CA1 neurons, PP1 reduces synaptic transmission during the induction of some forms of long-term depression (LTD) of synaptic transmission (Morishita et al., 2001). This could be one mechanism by which PP1 facilitates extinction of conditioned taste aversion in rats (Oberbeck et al., 2010) and promotes memory decline during water maze tasks in mice (Genoux et al., 2002). PP1 also constrains learning during short-interval (i.e., massed) associative training in mice (Genoux et al., 2002) and *H.c.* (Muzzio et al., 1999). To our knowledge, neither AA nor 12-LOX metabolites have been implicated in extinction learning in other (vertebrate) systems. However, related fatty acids in the endocannabinoid signaling system are believed to be important contributors to extinction. Cannabinoid (CB) receptors and their endogenous ligands, endocannabinoids, have been reported to participate in extinction of memory for aversive events in rats (Marsicano et al., 2002; Cannich et al., 2004). Despite the detection of endocannabinoids in *Aplysia* ganglia (Di Marzo et al., 1999), and other invertebrates (e.g., leech, Stefano et al., 1997), their existence in other invertebrate species is still debated (McPartland et al., 2006), and their role in learning has yet to be determined. Nevertheless, it is possible that AA/LOX-metabolites and associated ion channels (TRP and K^+^ channels) may be invertebrate evolutionary precursors to the endocannabinoid system of vertebrates, and play similar functional roles.

A final point concerns the fact that *in vitro* extinction reduced *Paired* B cell spiking below the levels of *Untrained* and *Random* control cells. If a simple reversal/erasure of the pairing-produced changes in cell excitability were occurring, then one might expect the spike frequencies of *Paired* cells to be indistinguishable from control cells during later LSs. However, *in vitro* extinction produced a pairing-specific decrease in spiking that was clearly lower than control cells by the 30th LS. The lower spike frequency of *Paired* cells is reminiscent of the persistent reductions in B cell spiking produced by CI learning (Britton and Farley, 1999). In conjunction with the common involvement of PP1 and AA/12(S)-HPETE in both CI and extinction, the *in vitro* results suggest that extinction may reflect “new” CI-like learning, although it is unclear whether the *in vitro*-produced reductions in spike frequency persist for 24 h. The view that the original molecular substrates of associative memories are completely “erased,” and the nervous system returned to its original *de novo* state by extinction training, may be overly simplistic. A more nuanced view of cellular and behavioral erasure effects by extinction might be that a new, possibly dynamic, equilibrium (between opposing original acquisition, “new” extinction learning, and erasure processes) is established following extinction training. Therefore, the original associatively-acquired behavioral changes could appear absent due to overall circuit level alterations, but changes at the scale of individual neurons (and subcellular alterations) might persist and reflect varying combinations of old and new learning mechanisms. Whether extinction in *H.c.* produces a complete erasure of the original associative memory might depend on the level of analysis (i.e., behavior, circuit, or individual neuron). Although it is a convenient heuristic to organize research efforts around mutual exclusive categories of extinction learning (i.e., erasure or new learning), this tendency could be overly simplistic and should not blind us to the inherit complexity of extinction learning.

### Conflict of interest statement

The authors declare that the research was conducted in the absence of any commercial or financial relationships that could be construed as a potential conflict of interest.

## References

[B1] Alkon D. L. Epstein H. Kuzirian A. Bennett M. C. Nelson T. J. (2005). Protein synthesis required for long-term memory is induced by PKC activation on days before associative learning. Proc. Natl. Acad. Sci. U.S.A. 102, 16432–16437. 10.1073/pnas.0508001102 16258064 PMC1283453

[B2] Alkon D. L. Sakakibara M. Forman R. Harrigan J. Lederhendler I. Farley J. (1985). Reduction of two voltage-dependent K^+^ currents mediates retention of a learned association. Behav. Neural Biol. 44, 278–300. 10.1016/S0163-1047(85)90296-1 4062781

[B3] Bader C. R. Baumann F. Bertrand D. (1976). Role of intracellular calcium and sodium in light adaptation in the retina of the honey bee drone (*Apis mellifera*). J. Gen. Physiol. 67, 475–491. 10.1085/jgp.67.4.475 818341 PMC2214920

[B4] Baumgärtel K. Genoux D. Welzl H. Tweedie-Cullen R. Y. Koshibu K. Liningstone-Zatchej M.. (2008). Control of the establishment of aversive memory by calcineurin and Zif268. Nat. Neurosci. 11, 572–578. 10.1038/nn.2113 18425121

[B5] Blackwell K. Farley J. (2009). Hermissenda. Scholarpedia 3, 4090–4095. 10.4249/scholarpedia.4090

[B6] Bouton M. E. (1994). Conditioning, remembering, and forgetting. J. Exp. Psychol. Anim. Behav. Process. 20, 219–231. 10.1037/0097-7403.20.3.219

[B7] Braha O. Dale N. Hochner B. Klein M. Abrams T. W. Kandel E. R. (1990). Second messengers involved in the two processes of presynaptic facilitation that contribute to sensitization and dishabituation in Aplysia sensory neurons. Proc. Natl. Acad. Sci. U.S.A. 87, 2040–2044. 10.1073/pnas.87.5.2040 2155432 PMC53621

[B8] Britton G. Farley J. (1999). Behavioral and neural bases of non-coincidence learning in *Hermissenda*. J. Neurosci. 19, 9126–9132. 10516330 10.1523/JNEUROSCI.19-20-09126.1999 PMC6782772

[B9] Cannich A. Wotjak C. T. Kamprath K. Hermann H. Lutz B. Marsicano G. (2004). CB1 cannabinoid receptors modulate kinase and phosphatase activity during extinction of conditioned fear in mice. Learn. Mem. 11, 625–632. 10.1101/lm.77904 15466318 PMC523082

[B10] Cavallo J. S. Hamilton B. Farley J. (2014). Behavioral and neural bases of extinction learning in *Hermissenda*. Front. Behav. Neurosci. 8:277. 10.3389/fnbeh.2014.00277 25191236 PMC4137458

[B11] Ceulemans H. Bollen M. (2004). Functional diversity of protein phosphatase-1, a cellular economizer and reset button. Physiol. Rev. 84, 1–39. 10.1152/physrev.00013.2003 14715909

[B12] Chad J. E. Eckert R. (1986). An enzymatic mechanism for calcium current inactivation in dialysed helix neurons. J. Physiol. 378, 31–51. 2432251 10.1113/jphysiol.1986.sp016206 PMC1182851

[B13] Chyb S. Raghu P. Hardie R. C. (1999). Polyunsaturated fatty acids activate the Drosophila light-sensitive channels TRP and TRPL. Nature 397, 255–259. 10.1038/16703 9930700

[B14] Clem R. L. Huganir R. L. (2010). Calcium-permeable AMPA receptor dynamics mediate fear memory erasure. Science 330, 1108–1112. 10.1126/science.1195298 21030604 PMC3001394

[B15] Cohen P. (1989). The structure and regulation of protein phosphatases. Annu. Rev. Biochem. 58, 453–508. 10.1146/annurev.bi.58.070189.002321 2549856

[B16] Crow T. (2004). Pavlovian conditioning of *Hermissenda*: current cellular, molecular, and circuit perspectives. Learn. Mem. 11, 229–238. 10.1101/lm.70704 15169851

[B17] Crow T. Forrester J. (1990). Inhibition of protein synthesis blocks long-term enhancement of generator potentials produced by one-trial *in vivo* conditioning in Hermissenda. Proc. Natl. Acad. Sci. U.S.A. 87, 4490–4494. 10.1073/pnas.87.12.4490 2352932 PMC54141

[B18] Di Marzo V. De Petrocellis L. Bisogno T. Melck D. (1999). Metabolism of anandamide and 2-arachidonoylglycerol: an historical overview and some recent developments. Lipids 34, S319–S325. 10.1007/BF02562332 10419192

[B19] Duerson K. White R. E. Jiang F. Schonbrunn A. Armstrong D. L. (1996). Somatostatin stimulates BK Ca channels in rat pituitary tumor cells through lipoxygenase metabolites of arachidonic acid. Neuropharmacology 35, 949–961. 10.1016/0028-3908(96)00131-1 8938725

[B20] Endo S. Critz S. D. Byrne J. H. Shenolikar S. (1995). Protein phosphatase-1 regulates outward K^+^ currents in sensory neurons of *Aplysia californica*. J. Neurochem. 64, 1833–1840. 10.1046/j.1471-4159.1995.64041833.x 7891112

[B21] Epstein H. T. Child F. M. Kuzirian A. M. Alkon D. L. (2003). Time windows for effects of protein synthesis inhibitors on Pavlovian conditioning in *Hermissenda*: behavioral aspects. Neurobiol. Learn. Mem. 79, 127–131. 10.1016/S1074-7427(02)00020-5 12591220

[B22] Farley J. (1987a). Contingency learning and causal detection in *Hermissenda*: I. Behavior. Behav. Neurosci. 101, 13–27. 10.1037/0735-7044.101.1.13 3828050

[B23] Farley J. (1987b). Contingency learning and causal detection in *Hermissenda*: II. Cellular mechanisms. Behav. Neurosci. 101, 28–56. 10.1037/0735-7044.101.1.28 2435301

[B24] Farley J. (1988a). Associative training results in persistent reductions in a calcium-activated potassium current in *Hermissenda* Type B photoreceptors. Behav. Neurosci. 102, 784–801. 10.1037/0735-7044.102.5.784

[B25] Farley J. (1988b). Causal detection in a mollusc: cellular mechanisms of predictive coding, associative learning and memory, in Harvard Symposium on Quantitative Analyses of Behavior: Vol. 7, Biological Determinants of Reinforcement, eds Commons M. Church R. Stellar J. Wagner A. (New Jersey, NJ: Lawrence Erlbaum Associates, Inc.), 207–248

[B26] Farley J. Alkon D. L. (1980). Neural organization predicts stimulus specificity for a retained associative behavioral change. Science 210, 1373–1375. 10.1126/science.7434033 7434033

[B27] Farley J. Alkon D. L. (1982). Associative neural and behavioral change in *Hermissenda*: consequences of nervous system orientation for light and pairing specificity. J. Neurophysiol. 48, 785–807. 6290619 10.1152/jn.1982.48.3.785

[B28] Farley J. Alkon D. L. (1987). *In vitro* associative conditioning of *Hermissenda*: cumulative depolarization of Type B photoreceptors and short-term associative behavioral changes. J. Neurophysiol. 57, 1639–1668. 3598626 10.1152/jn.1987.57.6.1639

[B29] Farley J. Auerbach S. (1986). Protein kinase C activation induces conductance changes in *Hermissenda* photoreceptors like those seen in associative learning. Nature 319, 220–223. 10.1038/319220a0 2418358

[B30] Farley J. Richards W. G. Ling L. J. Liman E. Alkon D. L. (1983). Membrane changes in a single photoreceptor cause associative learning in *Hermissenda*. Science 221, 1201–1203. 10.1126/science.6612335 6612335

[B31] Farley J. Schuman E. (1991). Protein kinase C inhibitors prevent induction and continued expression of cell memory in *Hermissenda* type B photoreceptors. Proc. Natl. Acad. Sci. U.S.A. 88, 2016–2020. 10.1073/pnas.88.5.2016 2000409 PMC51157

[B32] Fruman D. Klee C. Bierer B. Burakoff S. (1992). Calcineurin phosphatase activity in T lymphocytes is inhibited by FK 506 and cyclosporin A. Proc. Natl. Acad. Sci. U.S.A. 89, 3686–3690. 10.1073/pnas.89.9.3686 1373887 PMC525555

[B33] Genoux D. Haditsch U. Knobloch M. Michalon A. Storm D. Mansuy I. M. (2002). Protein phosphatase 1 is a molecular constraint on learning and memory. Nature 418, 970–975. 10.1038/nature00928 12198546

[B34] Goh Y. Lederhendler I. Alkon D. L. (1985). Input and output changes of an identified neural pathway are correlated with associative learning in *Hermissenda*. J. Neurosci. 5, 536–543. 3973682 10.1523/JNEUROSCI.05-02-00536.1985 PMC6565194

[B35] Grover L. M. Farley J. (1987). Temporal order sensitivity of associative learning in *Hermissenda*: behavior and neural correlates. Behav. Neurosci. 101, 658–675. 10.1037/0735-7044.101.5.658 3675844

[B36] Havekes R. Nijholt I. M. Visser A. K. Eisel U. L. Van der Zee E. A. (2008). Transgenic inhibition of neuronal calcinuerin activity in the forebrain facilitates fear conditioning, but inhibits the extinction of contextual fear. Neurobiol. Learn. Mem. 89, 595–598. 10.1016/j.nlm.2007.08.003 17884610

[B37] Huai Q. Kim H. Y. Liu Y. Zhao Y. Mondragon A. Liu J. O.. (2002). Crystal structure of calcineurin–cyclophilin–cyclosporin shows common but distinct recognition of immunophilin–drug complexes. Proc. Natl. Acad. Sci. U.S.A. 99, 12037–12042. 10.1073/pnas.192206699 12218175 PMC129394

[B38] Huang H. Farley J. (2001). PP1 Inhibitors depolarize *Hermissenda* photoreceptors and reduce K^+^ currents. J. Neurophysiol. 86, 1297–1311. 11535678 10.1152/jn.2001.86.3.1297

[B39] Ichinose M. Endo S. Critz S. D. Shenolikar S. Byrne J. H. (1990). Microcystein-LR, a potent protein phosphatase inhibitor, prolongs the serotonin- and cAMP-induced currents in sensory neurons of *Aplysia californica*. Brain Res. 533, 137–140. 10.1016/0006-8993(90)91806-R 1964827

[B40] Ito E. Oka K. Collin C. Schreurs B. G. Sakakibara M. Alkon D. L. (1994). Intracellular calcium signals are enhanced for days after Pavlovian conditioning. J. Neurochem. 62, 1337–1344. 10.1046/j.1471-4159.1994.62041337.x 8133264

[B41] Jin I. Huang H. Smith B. Farley J. (2009). Protein tyrosine kinase involvement in learning-produced changes in *Hermissenda* Type B photoreceptors. J. Neurophysiol. 102, 3573–3595. 10.1152/jn.90732.2008 19812284 PMC2804426

[B42] Kita A. Matsunaga S. Takai A. Kataiwa H. Wakimoto T. Fusetani N.. (2002). Crystal structure of the complex between calyculin A and the catalytic subunit of protein phosphatase 1. Structure 10, 715–724. 10.1016/S0969-2126(02)00764-5 12015153

[B43] Lederhendler I. I. Gart S. Alkon D. L. (1986). Classical conditioning of *Hermissenda*: origin of a new response. J. Neurosci. 6, 1325–1331. 3711982 10.1523/JNEUROSCI.06-05-01325.1986 PMC6568562

[B44] Lester D. S. Collin C. Etcheberrigaray R. Alkon D. L. (1991). Arachidonic acid and diacylglycerol act synergistically to activate protein kinase C *in vitro* and *in vivo*. Biochem. Biophys. Res. Commun. 179, 1522–1528. 10.1016/0006-291X(91)91745-X 1930192

[B45] Lin C. Yeh S. Leu T. Chang W. Wang S. Gean P. (2003). Identification of calcineurin as a key signal in the extinction of fear memory. J. Neurosci. 23, 1574–1579. 12629159 10.1523/JNEUROSCI.23-05-01574.2003 PMC6741970

[B46] Lindvall M. K. Pihko P. M. Koskinen A. M. (1997). The binding mode of calyculin A to protein phosphatase-1, a novel spiroketal vector model. J. Biol. Chem. 272, 23312–23316. 10.1074/jbc.272.37.23312 9287341

[B47] Lisman J. (1989). A mechanism for the Hebb and anti-Hebb processes underlying learning and memory. Proc. Natl. Acad. Sci. U.S.A. 86, 9574–9578. 10.1073/pnas.86.23.9574 2556718 PMC298540

[B48] Lisman J. Brown J. (1972). The effects of intracellular iontophoretic injection of calcium and sodium ions on the light response of *Limulus* ventral photoreceptors. J. Gen. Physiol. 59, 701–719. 10.1085/jgp.59.6.701 5025746 PMC2203200

[B49] Mao S. C. Hsiao Y. H. Gean P. W. (2006). Extinction training in conjunction with partial agonist of the glycine site on the NMDA receptor erases memory trace. J. Neurosci. 26, 8892–8899. 10.1523/JNEUROSCI.0365-06.2006 16943544 PMC6675349

[B50] Maren S. Chang C. H. (2006). Recent fear is resistant to extinction. Proc. Natl. Acad. Sci. U.S.A. 21, 18020–18025. 10.1073/pnas.0608398103 17090669 PMC1693865

[B51] Marks F. Furstenberger G. (1999). Prostaglandins, Leukotrienes and Other Eicosanoids: From Biogenesis to Clinical Application. Weinhein: Wiley-VCH

[B52] Marsicano G. Wotjak C. T. Azad S. C. Bisogno T. Rammes G. Grazia Cascio M. (2002). The endogenous cannabinoid system controls extinction of aversive memories. Nature 418, 530–534. 10.1038/nature00839 12152079

[B53] Marton M. J. DeRisi J. L. Bennett H. A. Iyer V. R. Meyer M. R. Roberts C. J.. (1998). Drug target validation and identification of secondary drug target effects using DNA microarrays. Nat. Med. 4, 1293–1301. 10.1038/3282 9809554

[B54] McPartland J. M. Agraval J. Gleeson D. Heasman K. Glass M. (2006). Cannabinoid receptors in invertebrates. J. Evol. Biol. 19, 366–373. 10.1111/j.1420-9101.2005.01028.x 16599912

[B55] Meves H. (2008). Arachidonic acid and ion channels: an update. Br. J. Pharmacol. 155, 4–16. 10.1038/bjp.2008.216 18552881 PMC2527843

[B56] Monfils M. H. Cowansage K. K. Kall E. LeDoux J. E. (2009). Extinction-reconsolidation boundaries: key to persistent attenuation of fear memories. Science 324, 951–955. 10.1126/science.1167975 19342552 PMC3625935

[B57] Morishita W. Connor J. H. Xia H. Quinlan E. M. Shenolikar S. Malenka R. C. (2001). Regulation of synaptic strength by protein phosphatase 1. Neuron 32, 1133–1148. 10.1016/S0896-6273(01)00554-2 11754843

[B58] Mulkey R. M. Endo S. Shenolikar S. Malenka R. C. (1994). Involvement of calcineurin/inhibitor 1 phosphatase cascade in hippocampus long-term depression. Nature 369, 486–488. 10.1038/369486a0 7515479

[B59] Muzzio I. A. Gandhi C. C. Manyam U. Pesnell A. Matzel L. D. (2001). Receptor-stimulated phospholipase A(2) liberates arachidonic acid and regulates neuronal excitability through protein kinase C. J. Neurophysiol. 85, 1639–1647. 11287487 10.1152/jn.2001.85.4.1639

[B60] Muzzio I. A. Ramirez R. R. Talk A. C. Matzel L. D. (1999). Interactive contributions of intracellular calcium and protein phosphatases to massed-trails learning deficits in *Hermissenda*. Behav. Neurosci. 113, 103–117. 10.1037/0735-7044.113.1.103 10197910

[B61] Myers K. M. Davis M. (2002). Behavioral and neural analysis of extinction. Neuron 36, 567–584. 10.1016/S0896-6273(02)01064-4 12441048

[B62] Myers K. M. Ressler K. J. Davis M. (2006). Different mechanisms of fear extinction dependent on length of time since fear acquisition. Learn. Mem. 13, 216–223. 10.1101/lm.119806 16585797 PMC1409828

[B63] Oberbeck D. L. McCormack S. Houpt T. A. (2010). Intra-amygdalar okadaic acid enhances conditioned taste aversion learning and CREB phosphorylation in rats. Brain Res. 1348, 84–94. 10.1016/j.brainres.2010.06.029 20599840 PMC2931335

[B64] Orgad S. Dudai Y. Cohen P. (1987). The protein phosphatases of *Drosophila melanogaster* and their inhibitors. Eur. J. Biochem. 164, 31–38. 10.1111/j.1432-1033.1987.tb10988.x 3030753

[B65] Pavlov I. P. (1927). Conditioned Reflexes. London: Oxford University Press

[B66] Pondhaven P. Cohen P. (1987). Identification of protein phosphatases-1 and 2A and inhibitor-2 in oocytes of the starfish *Asterias rubens* and *Marthasterias glacialis*. Eur. J. Biochem. 167, 135–140. 10.1111/j.1432-1033.1987.tb13314.x 3040398

[B67] Rescorla R. A. Cunningham C. L. (1978). Recovery of the US representation over time during extinction. Learn. Motiv. 9, 373–391. 10.1016/0023-9690(78)90001-2

[B68] Rescorla R. A. Heth C. D. (1975). Reinstatement of fear to an extinquished conditioned stimulus. J. Exp. Psychol. Anim. Behav. Process. 1, 88–96. 10.1037/0097-7403.1.1.88 1151290

[B69] Resjö S. Oknianska A. Zolnierowicz S. Manganiello V. Degerman E. (1999). Phosphorylation and activation of phosphodiesterase type 3B (PDE3B) in adipocytes in response to serine/threonine phosphatase inhibitors: deactivation of PDE3B *in vitro* by protein phosphatase type 2A. J. Biochem. 341, 839–845. 10.1042/0264-6021:3410839 10417351 PMC1220425

[B70] Richards W. Farley J. (1987). Motor correlates of phototaxis and associative learning in *Hermissenda crassicornis*. Brain Res. Bull. 19, 175–189. 10.1016/0361-9230(87)90083-9 3664278

[B71] Richards W. Farley J. Alkon D. (1984). Extinction of associative learning in *Hermissenda*: behavior and neural correlates. Behav. Brain Res. 14, 161–170. 10.1016/0166-4328(84)90185-2 6525240

[B72] Robbins S. J. (1990). Mechanisms underlying spontaneous recovery in autoshaping. J. Exp. Psychol. Anim. Behav. Process. 16, 235–249. 10.1037/0097-7403.16.3.235

[B73] Rühlmann A. Nordheim A. (1997). Effects of the immunosuppressive drugs CsA and FK506 on intracellular signaling and gene regulation. Immunobiology 198, 192–206. 10.1016/S0171-2985(97)80040-X 9442391

[B74] Sakakibara M. Alkon D. L. Kouchi T. Inoue H. Yoshioka T. (1994). Induction of photoresponse by the hydrolysis of polyphosphoinositides in the *Hermissenda* Type B photoreceptor. Biochem. Biophys. Res. Commun. 202, 299–306. 10.1006/bbrc.1994.1927 7518677

[B75] Sakakibara M. Alkon D. L. Neary J. T. Heldman E. Gould R. (1986). Inositol trisphosphate regulation of photoreceptor membrane currents. Biophys. J. 50, 797–803. 10.1016/S0006-3495(86)83520-2 3491632 PMC1329804

[B76] Sangha S. Scheibenstock A. Morrow R. Lukowiak K. (2003). Extinction requires new RNA and protein synthesis and the soma of the cell right pedal dorsal 1 in *Lymnaea stagnalis*. J. Neurosci. 23, 9842–9851. 14586013 10.1523/JNEUROSCI.23-30-09842.2003 PMC6740901

[B77] Schiller D. Monfils M. H. Raio C. M. Johnson D. C. LeDoux J. E. Phelps E. A. (2010). Preventing the return of fear in humans using reconsolidation update mechanisms. Nature 463, 49–53. 10.1038/nature08637 20010606 PMC3640262

[B78] Sharma K. Bagnall M. W. Sutton M. A. Carew T. J. (2003). Inhibition of calcineurin facilitates the induction of memory for sensitization in *Aplysia*: requirement of mitogen-activated protein kinase. Proc. Natl. Acad. Sci. U.S.A. 100, 4861–4866. 10.1073/pnas.0830994100 12672952 PMC153646

[B79] Shi Y. (2009). Serine/threonine phosphatases: mechanism through structure. Cell 139, 468–484. 10.1016/j.cell.2009.10.006 19879837

[B80] Shuttleworth T. J. Thompson J. L. Mignen O. (2004). ARC channels: a novel pathway for receptor-activated calcium entry. Physiology 19, 355–361. 10.1152/physiol.00018.2004 15546853

[B81] Silva A. J. Kogan J. H. Frankland P. W. Kida S. (1998). CREB and memory. Annu. Rev. Neurosci. 21, 127–148. 10.1146/annurev.neuro.21.1.127 9530494

[B82] Stafstrom-Davis C. A. Ouimet C. C. Feng J. Allen P. B. Greengard P. Houpt T. A. (2001). Impaired conditioned taste aversion learning in spinophilin knockout mice. Learn. Mem. 8, 272–278. 10.1101/lm.42101 11584074 PMC311386

[B83] Stefano G. B. Salzet B. Rialas C. M. Pope M. Kustka A. Neenan K.. (1997). Identification and characterization of the leech CNS cannabinoid receptor: coupling to nitric oxide release. Brain Res. 753, 219–224. 10.1016/S0006-8993(96)01484-9 9125406

[B84] Stemmer P. M. Klee C. B. (1994). Dual calcium ion regulation of calcineurin by calmodulin and calcineurin B. Biochemistry 33, 6859–6866. 10.1021/bi00188a015 8204620

[B85] Sugita S. Baxter D. A. Byrne J. H. (1994). Activators of protein kinase C mimic serotonin-induced modulation of a voltage-dependent potassium current in pleural sensory neurons of *Aplysia*. J. Neurophysiol. 72, 1240–1249. 7807208 10.1152/jn.1994.72.3.1240

[B86] Talk A. C. Muzzio I. A. Matzel L. D. (1997). Phospholipases and arachidonic acid contribute independently to sensory transduction and associative neuronal facilitation in *Hermissenda* type B photoreceptors. Brain Res. 751, 196–205. 10.1016/S0006-8993(96)01397-2 9099806

[B87] Walker T. L. Campodonico J. Cavallo J. S. Farley J. (2010). AA/12-lipoxygenase signaling contributes to inhibitory learning in *Hermissenda* type B photoreceptors. Front. Behav. Neurosci. 4:50. 10.3389/fnbeh.2010.00050 20802857 PMC2928666

